# Phase Transition and an Instanton Model of Geiger-Mode Pulses in Single-Photon Avalanche Diodes

**DOI:** 10.3390/s26196170

**Published:** 2026-09-29

**Authors:** Yutaka Hirose, Akito Inoue

**Affiliations:** Panasonic Industry Co., Ltd., Kadoma 571-8506, Japan; inoue.akito@jp.panasonic.com

**Keywords:** single-photon avalanche diode (SPAD), quenching, CMOS image sensor (CIS), Impact Ionization Transit-Time (IMPATT) Diode, Lienard equation, phase transition, symmetry breaking, instanton, path integral, superconductivity

## Abstract

**Highlights:**

**What are the main findings?**

- A model in which various operational modes of an avalanche diode are regarded as “phases” of a unified dynamical system described by a Lienard-type equation is developed.
- The role of an excess voltage with respect to the breakdown voltage is found to introduce an initial “symmetry-breaking” into a symmetric available energy profile of the breakdown voltage condition, whereas increase in a quenching resistor value causes a “symmetry-breaking instability” at the breakdown voltage exhibiting a phase transition to a Geiger-mode pulse generation.

**What are the implications of the main findings?**

- Associated with the phase transition, a quantum mechanical model in which a passive quenching process of a Geiger-mode pulse is described as a pairing of an instanton (discharge phase) and an anti-instanton (recharge phase) bonded by a carrier extinction event.
- The developed phenomenology defined in the time domain is found to have similarities to that of superconductivity described in the space or momentum coordinate.

**Abstract:**

A theoretical study categorizing the modes or the “phase” of the carrier dynamics in a single-photon avalanche diode is presented. For the basic model described by a nonlinear Lienard equation, a Lagrangian, a Hamiltonian, and a Lyapunov function are identified. From the energetics studies, variation in the phases is found to be described by the profile (symmetry) change in the available energy induced by change in the quenching resistance value. Each phase is found to correspond to an operational mode of the device; a diode biased at the breakdown voltage, Impact Ionization Transit-Time Diode, and single-photon avalanche diode. Bifurcation points of the Lienard equation are regarded as phase transition points. The Geiger-mode pulse phase is found to emerge with a symmetry-breaking instability in the available energy profile beyond a threshold value of the quenching resistance. Utilizing energy conservation, high-speed transition processes of discharge and a recharge periods are, respectively, formulated as an instanton and an anti-instanton, particles highly localized in time, by quantum mechanical path integrals. A successfully quenched Geiger-mode pulse is described by an instanton–anti-instanton pair bonded by a key condition of the carrier extinction effect. An analogy of the phenomenology to superconductivity is discussed.

## 1. Introduction

Single-photon avalanche diodes (SPADs) enable one to detect optical power in units of individual photons by generating a gigantic avalanche-multiplied current, Geiger-mode (GM), pulse triggered by a single photocarrier and revolutionize high-sensitivity optical detection technologies [1,2,3]. In particular, in sync with continuously down-scaling silicon complementary metal-oxide semiconductor (CMOS) technology, developments of SPAD-based CMOS image sensors (SPAD-CISs) with pixel-level photon counting functions are in rapid progress [4,5,6]. Recently, mega-pixel imagers [7,8], small-pixel (3 μm) imagers [9], and imagers with a stacked backside-illumination architecture [9,10] have been reported. Promising applications are time-of-flight ranging systems [11], high dynamic range imaging [12], low-light-level imaging [13], and biological fluorescence imagers [14,15].

Two technological foundations of SPAD-CISs are (i) the realization of the idling state under a bias condition in excess of the breakdown voltage (BV); and (ii) the establishment of “quenching” the avalanche current within a short period (a few hundred pico-seconds) and of recovering the idling state. As an image sensor’s fundamental performances, item (i) defines a base-line (zero-photon) image while item (ii) guarantees a reset operation. Regarding (i), the value of the excess voltage (EV) defines an avalanche triggering probability (ATP) [16], an amount and a period of discharging prestored depletion charge by the avalanche effect. To set up this condition, we must take into account not only guaranteed zero carrier injection to prevent a dark count pulse, but also instabilities due to the application of an EV leading to fluctuation due to micro-plasmas [17] and phase difference between carriers and voltage [18]. Regarding (ii), the quenching action is realized by a negative feedback effect of quenching circuits consisting of a resistor–capacitor network in series with the SPAD [3,19]. The quenching resistance (QR) determines the fall-time of a recharge period. Empirically, a relatively high value (about 50 kΩ) of a QR that allows discharged carriers to be stored on a series capacitor was found to be a necessary criterion for successful quenching [3]. Thus, design of a SPAD pixel circuit is essentially tuning the values of EV and QR to optimize the two opposite effects; the amount and period of signal generation (discharge by EV) and the speed of quenching (recharge by QR). Controlling transition from the discharge to the recharge period is crucial for successful quenching. In this respect, various circuit models [20,21,22] are demonstrated to be practically of high value.

However, a physical theory that presents a clear relation between EV and QR is scarce. Only recently, a nonlinear carrier dynamics model based on a Lienard equation (LE) is shown to lead to a critical condition for QR explicitly depending on EV [23]. Separated by bifurcation points of the LE, carrier dynamics of an SPAD were categorized into three different regions; overdamped (low QR values), spiral-stable (higher QR values), and sink-node (infinite QR value) regimes. For successful quenching, it was also found that carrier extinction (CE) previously found as a quantization condition [24] was necessary in addition to the above criterion. The theory was also found to be consistent with the characteristics of steady state IMPATT diodes’ operational modes [25]. These results point to a possibility of unifying the description of the carrier dynamics in the avalanche devices by regarding the categorized carrier dynamics modes as phases of an identical object. In particular, the divergence of the carrier number at the BV hints at the existence of a kind of phase transition between these phases, just like appearances of an infinite conductivity below critical temperature in a superconductor ([26], pp. 110–147).

In this paper, we analytically derive such a viewpoint of a phase transition model for an SPAD from the above LE-based formulation. With the starting LE, a Lagrangian and a Hamiltonian are identified. From the Hamiltonian, energy conservation is also confirmed. A Lyapunov function giving an asymptotic stability condition is found. Using an available energy profile (AEP) derived from the Lagrangian, a phase transition model analogous to the free energy in the Ginzburg–Landau theory ([26], pp. 110–147) is presented. Operational modes of an avalanche device are classified into different phases represented by the AEP as a function of the QR value, R. A significant role of EV is found; with no EV (denoted as $V_{ex}$), a “symmetric” AEP phase corresponding to a DC-biased steady state results. Application of a finite $V_{ex}$ is found to break the symmetry at the initial condition as indicated by the occurrence of a double well in the energy profile corresponding to instability near BV. However, in a low R (R→0) limit, the system stays in the overdamped state with the BV as a stable equilibrium point; after starting an avalanche breakdown, the system returns to the steady symmetric state. Thus, the system is regarded as being in a quasi-symmetric phase corresponding to the operational mode of IMPATT diodes. On the other hand, an instability at BV is found to start at the onset of transition from an overdamped to a spirally stable state by continuous increase in R. The available energy at BV is no longer a local minimum. Ultimately, in a R→∞ limit, the energy profile exhibits a complete double well. A GM-pulse under a successful quenching condition is identified as a round-trip-transition between the local minima of the AEP represented as a double well. The entire phase diagram is constructed on a 2D $R-V_{ex}$ plane. Then, using the identified Lagrangian (Hamiltonian) and the classical solution of the LE, a quantum mechanical “instanton” model is developed by a Feynman path integral scheme under a stationary phase approximation condition ([27], pp. 210–219). Green’s functions arising from transitions between local minima of the available energy’s double well are derived. In particular, the avalanche discharge period and the recharge period during quenching are, respectively, identified as “instanton” and “anti-instanton” because they have opposite signs of momentum or direction of transition ([27], pp. 210–219). A wave function describing a photon counting process is constructed as successive quenching pulses comprising a product of instanton–anti-instanton pairs. Finally, there are similarities and differences in the SPAD carrier dynamics with the phenomenology of superconductivity. An overview of the analysis flow is illustrated in Figure 1.

## 2. Summary of the Basic Model

We start with summarizing derivation of the basic model based on a LE [23]. We consider a one-dimensional SPAD with a p-i-n structure where the i-region is a multiplication region (MR). A circuit model where a QR, R, is connected to the cathode of the SPAD is illustrated in Figure 2a. A bias voltage, $V_{0}$, is higher than the BV of the SPAD, $V_{BD}$, by a small EV, $V_{ex}$; $0<V_{ex}\ll \;V_{BD}$ <${\;V}_{0}=V_{BD}+V_{ex}$. The anode of the SPAD is grounded. The capacitance, C, at the cathode represents the sum of the capacitances of the SPAD MR and stray components at the node. In Figure 2b, a band diagram of the one-dimensional model of the SPAD overlapped on an equivalent circuit model is illustrated. The main assumptions are as follows:

(i) Inside the MR, an electric field is uniform, and the width of the MR is constant.
(ii) By impact ionization, carriers are generated at the center of the MR.
(iii) Inside the MR, carriers transport with their saturation velocity; $v_{e}$ for electrons and $v_{h}$ for holes.

Definitions of relevant parameters are listed in Table 1. The LE is derived from a drift-ionization model. First, we set up rate equations for electrons and holes in the MR as

(1) $$\frac{dn\left(t\right)}{dt}=\alpha \left(t\right)v_{e}n\left(t\right)+\beta \left(t\right)v_{h}p\left(t\right)-\frac{2v_{e}}{W}n\left(t\right),$$

(2) $$\frac{dp\left(t\right)}{dt}=\alpha \left(t\right)v_{e}n\left(t\right)+\beta \left(t\right)v_{h}p\left(t\right)-\frac{2v_{h}}{W}p\left(t\right).$$

We employ the Sze–Gibbons impact ionization coefficients of which time dependences are due to those of electric fields [28]; $\alpha \left(t\right)=\alpha_{0}\cdot \exp\left(-a/E(t)\right)$ for electrons and $\beta \left(t\right)=\beta_{0}\cdot \exp\left(-b/E(t)\right)$ for holes. Eliminating hole-related terms, and using the relations between $V\left(t\right)$ and the number of electrons, $N_{C}$, stored on the capacitance C,

(3) $$V\left(t\right)=V_{0}-q\cdot \frac{N_{C}\left(t\right)}{C}\;,$$

(4) $$\frac{dN_{C}(t)}{dt}=\frac{2v_{e}}{W}n\left(t\right)-\frac{N_{C}\left(t\right)}{R_{Q}\cdot C}$$

Then, V(t) is described by the following LE ((14) and (15) in [23]).

(5) $$\ddot{V}\left(t\right)+\frac{1}{RC}\dot{V}\left(t\right)-\frac{1}{\tau_{Q}}\frac{V\left(t\right)-V_{BD}}{V_{ex}}\dot{V}\left(t\right)+\frac{1}{RC}\frac{1}{\tau_{Q}}\frac{V_{0}-V\left(t\right)}{V_{ex}}\cdot \left(V\left(t\right)-V_{BD}\right)=0,$$

where $\tau_{Q}$ is a characteristic time constant of the avalanche process defined as

(6) $$\frac{1}{\tau_{Q}}\equiv \left(\alpha^{\prime }\left(E_{BD}\right)+\beta^{\prime }\left(E_{BD}\right)\right)\cdot \frac{v_{e}}{W}\cdot V_{ex}=\frac{V_{ex}}{\tau^{\prime }}$$

Here, $\alpha^{\prime }\left(E_{BD}\right)$ and $\beta^{\prime }\left(E_{BD}\right)$ are defined as $\alpha^{\prime }\left(E_{BD}\right)\equiv d\alpha /dE{|}_{E=E_{BD}}$ and $\beta^{\prime }\left(E_{BD}\right)\equiv d\beta /dE{|}_{E=E_{BD}}$. For later convenience, we rewrite (5) as follows:

(7) $$\ddot{\varphi }\left(t\right)+\frac{1}{\tau_{Q}}\left(\frac{\tau_{Q}}{RC}+\frac{\varphi \left(t\right)}{V_{ex}}\right)\cdot \dot{\varphi }\left(t\right)+\frac{1}{RC}\frac{1}{\tau_{Q}}\frac{\varphi \left(t\right)+V_{ex}}{V_{ex}}\cdot \varphi \left(t\right)=0,$$

where

(8) $$\varphi \left(t\right)\equiv V_{BD}-V\left(t\right).$$

The range of $\varphi \left(t\right)$ is $-V_{ex}\leq \varphi \left(t\right)\leq V_{ex}$ and a relational chart between $V_{0}$, $V_{BD}$, $V_{ex}$, $V\left(t\right)$, and $\varphi \left(t\right)$ is shown in Figure 2c.

Equations (5) or (7) was numerically evaluated previously [23]. $\varphi \left(t\right)$s’ values for different QR values are plotted in Figure 3a. With a small value of R (=500 Ω), $\varphi \left(t\right)$ quickly converges to 0, or $V\left(t\right)=V_{BD}$ without any overshoot nor oscillation exhibiting an overdamped oscillator character as plotted by an orange solid line. Because the final voltage is $V_{BD}$ with avalanche current flowing, quenching of this current is impossible in this regime. In this case, the final state, ($\varphi =0,\dot{\varphi }=0$), is characterized as a fixed point with a simple “sink node” character [29]. Once, the system enters into the “sink node”, there is no possibility of recovering the idling state, $\varphi =-V_{ex}$. With increase in R, the overshoot effect becomes significant as shown by the green (R=10 kΩ) and blue (R=100 kΩ) solid lines. $V\left(t\right)$ fluctuates around and eventually converges to φ = 0. In this regime, the final state, ($\varphi \left(t\right)=0,\dot{\varphi }=0$), is categorized as a fixed point with a “stable spiral” character [28] defined on the “continuous” variable $\varphi \left(t\right)$. From a trace-determinant analysis of the Jacobian matrix of (5), the bifurcation of the final fixed point, or the transition from the “sink node” to “stable spiral” character, occurs when R reaches the critical value, $R=R_{th}=\tau_{Q}/\left(4C\right)$ [23]. To successfully quench the avalanche current initially biased with an EV, it is necessary to have the QR value “higher than” $R_{th}$. In the present simulation, $R_{th}=550\;\Omega$. The significance of $R_{th}$ is discussed in Section 6. Yet, if we did not introduce a forced carrier extinction (CE) as defined by $n\left(t\right)=0$ whenever $n\left(t\right)<1$, even in this regime, quenching would not be successful due to ionization of the numerically allowed “fractional charge” of the “continuous” variable $n\left(t\right)$ [24]. Thus, a CE condition is necessary for a successful quenching [23,24]. With a relatively large R (=100 kΩ), and introduction of the CE condition, numerical calculation of (7) successfully reproduces a successfully quenched GM-pulse as plotted by a yellowish dotted line. It is noted that the necessary condition for a successful quenching is derived by a geometrical analysis as in [23].

(9) $$R>\frac{2t_{BD}}{C\cdot ln(2)}=\frac{2\tau_{Q}}{C\cdot ln(2)}\cdot \ln\left(\frac{CV_{ex}W}{qv_{s,e}\tau_{Q}}\right)\equiv R_{Q}$$

In a limit R→∞, $\varphi \left(t\right)$ shows a single discharge event represented by a sigmoid function with the time constant $\tau_{Q}$. Since in this case, no recharge through R is possible, the process ends at the lowest voltage “below” $V_{BD}$.

Corresponding trajectories in the phase space are plotted in Figure 3b with the same color lines. With the smallest R(=500 Ω), starting from the initial point (IP), i.e., ($\varphi =-V_{ex},\;\dot{\varphi }=0$), the trajectory follows a small parabolic curve ending at the final point (FP) of the origin. With the largest value of R(→∞), the trajectory follows a outermost parabolic curve with the maximum end point value of ${\varphi =V}_{ex}$ showing an overshoot with respect to $V_{BD}$ by the amount of $V_{ex}$. With a finite and relatively large valued R(→100 kΩ) satisfying (9), the trajectories exhibit spiral around the origin corresponding to the damped oscillation in Figure 3a. When the CE condition is met, the curve snaps back to the IP after passing a turning point (TP) with a maximum value of φ (close to $V_{ex}$) showing a successful quenching as plotted by a dotted yellowish line. In this case, the path from the TP to the IP is a recharge phase and follows a straight line indicating an exponential decay time constant formed by R and C. Reviewing (9), the quenching condition is regarded as the necessity of the quenching RC-time constant being longer than the avalanche lifetime, $\tau_{Q}$, multiplied by the specified coefficient. Typical values of $\tau_{Q}$ and $R_{Q}C$ time constants are, respectively, 10 ps and 300 ps. It is emphasized that $\tau_{Q}$ is a function of $V_{ex}$ defined as (6).

## 3. Classical Mechanical Properties of the Lienard Equation (LE)

In this section, we investigate classical or analytical mechanical properties of the LE (7). We construct a Lagrangian and a Hamiltonian of an LE described in a general form. After identifying a kinetic energy term and potential terms, the LE itself is derived from the Euler–Lagrange equation and from the Hamilton equation. Energy conservation is also confirmed. Then, applying these results, a Lyapunov function is constructed and an asymptotic stability condition is derived. It is shown that, for successful quenching, the asymptotic stability condition should be violated or “instability” is necessary.

### 3.1. Energy Terms, Lagrangian, Hamiltonian

A general form of an LE representing (7) is as follows;

(10) $$\ddot{x}+f\left(x\right)\cdot \dot{x}+g\left(x\right)\cdot x=0,$$

where x is a time-dependent variable and $f\left(x\right)$ and $g\left(x\right)$ are general functions of x. For a moment, we regard x as a general position coordinate of a particle with unit mass. Substituting $x=\varphi \left(t\right)$, and comparing (7) and (10), these two equations are identical if

(11) $$f\left(\varphi \left(t\right)\right)=\frac{1}{R\cdot C}+\frac{\varphi \left(t\right)}{\tau_{Q}\cdot V_{ex}},$$

(12) $$g\left(\varphi \left(t\right)\right)=\frac{1}{\tau_{Q}\cdot R\cdot C\cdot V_{ex}}\left(\varphi \left(t\right)+V_{ex}\right).$$

Then, $\dot{x}$ and $\ddot{x}$ are regarded as, respectively, the particle’s velocity and acceleration. Then, the kinetic energy of the particle, T, is defined as

(13) $$T\left(\dot{x}\right)=\frac{1}{2}{\left(\dot{x}\right)}^{2}.$$

By treating the second and the third terms of (10) as a total derivative of x, we obtain an effective potential term, V, as

(14) $$V\left(x\right)=\int_{x_{0}}^{x}f\left(y\right)\cdot \dot{y}\cdot dy+\int_{x_{0}}^{x}g\left(y\right)\cdot y\cdot dy,$$

where $x_{0}$ is an initial value of the variable x. Here, the word “effective” indicates that the potential contains all the relevant energy terms including dissipation. Such construction leads to a conservative force field, $\ddot{x}=-\partial V\left(x\right)/\partial x$, through the Lienard equation itself as pointed out in Appendix A. The first term of the right-hand side of (14) is an energy loss term proportional to the velocity $\left(\dot{y}\right)$ while the second term is a position-dependent potential term. Then, a Lagrangian is

(15) $$L\left(x,\;\dot{x}\right)=T\left(\dot{x}\right)-V\left(x\right).$$

We confirm that (15) is a proper Lagrangian giving (10) by taking the Euler–Lagrange equation as follows.

(16) $$\frac{d}{dt}\left(\frac{\partial L}{\partial \dot{x}}\right)-\frac{\partial L}{\partial x}=\ddot{x}+f\left(x\right)\cdot \dot{x}+g\left(x\right)\cdot x=0,$$

which satisfies the LE (10) as expected.

From the Lagrangian, we obtain a canonical momentum, p, as

(17) $$p\equiv \frac{\partial L}{\partial \dot{x}}=\dot{x}.$$

Then, the corresponding Hamiltonian, H, is obtained as

(18) $$H\left(x,\;\dot{x}\right)=p\cdot \dot{x}-L\left(x,\;\dot{x}\right)=T\left(\dot{x}\right)+V\left(x\right).$$

Taking the total time derivative, we confirm an energy conservation law by recovery of the original LE (10) as follows.

(19) $$\frac{dH}{dt}=\left(\ddot{x}+f\left(x\right)\cdot \dot{x}+g\left(x\right)\cdot x\right)\cdot \dot{x}=0.$$

From the conservation law, we obtain potential energy terms without explicit calculation of (14). It is also noted that (18) satisfies the Hamilton equation pair through the LE (10) as follows.

(20) $$\frac{\partial H}{\partial x}=-\ddot{x},\;\;\;\frac{\partial H}{\partial \dot{x}}=\dot{x}.$$

### 3.2. Lyapunov Function and a Necessary Condition for Quenching an SPAD

For a given dynamical system, with a fixed equilibrium point $x^{*}$, a finite function, $U(x,\dot{x})$, defined in a region satisfying the following conditions is defined as the Lyapunov function [30] of the system.

(21) $$x\neq x^{*},\;U\left(x,\;\dot{x}\right)\geq U\left(x^{*},\;{\dot{x}}^{*}\right),$$

(22) $$x\neq x^{*},\;\frac{dU\left(x,\;\dot{x}\right)}{dt}\;\leq 0.$$

If a Lyapunov function existed, then, the given fixed point would be proven to be an asymptotically stable point; $x\to x^{*}\;as\;t\to \infty$. For the general form LE (10), a Lyapunov function satisfying (21) and (22) can be obtained as follows. Let $x=x^{*}$ be a fixed point, and excluding the loss term from the Hamiltonian (18), we set

(23) $$U\left(x,\;\dot{x}\right)=\frac{1}{2}{\left(\dot{x}\right)}^{2}+\int_{x_{0}}^{x}g\left(y\right)\cdot y\cdot dy.$$

For a finite range of x, $U\left(x,\;\dot{x}\right)$ is assumed to be finite. Then, as long as (22) is met, (21) is automatically satisfied. As for (22), by taking the time derivative of U,

(24) $$\frac{dU}{dt}=\ddot{x}\cdot \dot{x}+g\left(x\right)\cdot x\cdot \dot{x}=-f\left(x\right)\cdot {\left(\dot{x}\right)}^{2},$$

where the second equality of the right-hand side is due to the LE (10). Therefore, for U to be a Lyapunov function, the following condition should be met,

(25) $$f\left(x\right)>0.$$

Thus, when (21) and (25) are satisfied, the constructed (23) is qualified as a Lyapunov function of the given LE (10).

Returning to our original LE, (7), (11), (12), since the initial value of $\varphi \left(t\right)$ is ${-V}_{ex}$, and the final (equilibrium point) value is 0, the condition (25) leads to $f\left({-V}_{ex}\right)=\frac{1}{R\cdot C}-\frac{1}{\tau_{Q}}>0$. Thus, we obtain the Lyapunov stable condition of (7) as

(26) $$R<\frac{\tau_{Q}}{C}\equiv R_{L}.$$

Equation (26) is the condition that the system is in a stable state; an SPAD initially biased with an excess bias voltage by $V_{ex}$ surely falls into the final point of the BV, $V_{BD}$, as the equilibrium (fixed) point ($x^{*}$). Any GM-pulse will not be quenched. This is consistent with the fact that $R_{L}$ is smaller than $R_{Q}$ (the necessary condition for a successful quenching) by an order of magnitude. In the range $R_{L}<R<R_{Q}$, the system is unstable. Neither a successful quenching nor convergence to the equilibrium point is guaranteed, possibly suffering from an uncontrollable fluctuation around $V_{BD}$. Restating the quenching condition of an SPAD, we need an R value far exceeding $R_{L}$ and at least larger than $R_{Q}$. The range specified by $R_{L}<R<R_{Q}$ acts as an interface range from the stable unquenchable regime to the quenchable unstable regime. It is also noted that the operational condition for IMPATT-type devices falls into the region specified by (26), avoiding the above interface region [23,24,31]. Thus, the operational conditions of avalanche-effect devices are set by the value of the series or QR. In the next section, we investigate the variation in the carrier dynamics or phase change induced by the change in R.

## 4. Phase Transition Model and Classical Instanton Solutions

In this section, we develop a phase transition model for the carrier dynamics of an avalanche diode described by (7). The kinetic energy term (11) is reinterpreted as an AEP which describes characteristics of a phase of the operational mode. A phase transition is induced by variation in the value of QR, (=R) in a similar way to that described in the Ginzburg–Landau theory ([26], pp. 110–147). The value of the QR is shown to play the role of temperature in the conventional GL theory. In the limiting cases of R, we find explicit analytical solutions of (7) corresponding to classical instanton solutions ([32], pp. 403–416). In the following, the voltage variable φ is treated as if it were an order parameter describing the free energy. Although such treatment is a formal one at this point, quantization conditions of φ discussed in Appendix A are a support for such treatment.

### 4.1. Low-Resistance (IMPATT Diode), Zero-V_ex_ (BV), and Negative-V_ex_ Regimes

We first take an R→0 limit in (7) leading to

(27) $$\dot{\varphi }+\frac{1}{\tau_{Q}\cdot V_{ex}}\cdot \varphi \cdot \left(\varphi +V_{ex}\right)=0,$$

where the index describing the time dependence of φ is omitted for a simple notation. As noted in the previous section, this condition on R falls into the IMPATT diode operational mode. Although the first term of (27) may be regarded as a velocity term of a pseudo-particle system ([27], pp. 457–466) as in the previous section, we reinterpret the physical meaning of the term according to the original setting of the SPAD circuit illustrated in Figure 2a. Since $\dot{\varphi }$ is a time derivative of the voltage of the capacitance storage node, $C\dot{\varphi }$ is the current due to discharging or charging of the capacitor flowing through the QR. Therefore, ${\left(C\dot{\varphi }\right)}^{2}$ is proportional to the power or the energy density available to the QR. Thus, for simplicity, we call the term $\frac{1}{2}{\left(\dot{\varphi }\right)}^{2}$ as the AEP and denote it $A\left(\varphi \right)$ obtained from (27) as

(28) $$A\left(\varphi \right)=\frac{1}{2}{\left(\dot{\varphi }\right)}^{2}=\frac{1}{2}\cdot \frac{1}{{\tau_{Q}}^{2}\cdot {V_{ex}}^{2}}\cdot \varphi^{2}\cdot {\left(\varphi +V_{ex}\right)}^{2}.$$

(29) is plotted as an orange line at the top graph of Figure 4. The maximum of $A\left(\varphi \right)$ is given by

(29) $$A\left(-\frac{1}{2}V_{ex}\right)=\frac{1}{32}\cdot \frac{{V_{ex}}^{2}}{{\tau_{Q}}^{2}}=\frac{1}{32}\cdot \frac{{V_{ex}}^{4}}{{\tau^{\prime }}^{2}},$$

where the relation (6) is taken into account for the second equality. The meaning of the above analysis becomes clear when we take a further limit of $V_{ex}\to 0$ in (28). Taking account of (6) in (28) leads to

(30) $$A\left(\varphi \right)=\frac{1}{2}\cdot \frac{1}{{\tau^{\prime }}^{2}}\cdot \varphi^{4},$$

where $\tau^{\prime }$ is defined in (6). (30) is an approximate available energy curve for zero $V_{ex}$ limit, i.e., BV-biased limit, and plotted in the top graph of Figure 4 with a light green color. The curve is “symmetrical” with respect to the origin and exhibits a profile identical to an anharmonic oscillator oscillating around the BV. The average capacitance storage node (anode of SPAD) voltage is steadily fixed (DC output) at $V_{BD}$. In contrast, the curve (28) is not symmetrical with respect to the origin with an extra minimum at $\varphi =-V_{ex}$. Thus, we interpret that application of an EV, $V_{ex}$, breaks the symmetry of the steady state AEP of (30), indicating a type of phase transition exhibiting a double well or a “bi-stability”. (29) is then regarded as the maximum energy contributed to instability. Yet, once φ transits to the FP, the system recovers the symmetric state of (30) since the FP is the stable fixed point. Thus, the low R region is regarded as a quasi-symmetric phase.

Returning to (27), this is a logistic equation with an exact solution,

(31) $$\varphi \left(t\right)=-\frac{V_{ex}\cdot \exp\left(-t/\tau_{Q}\right)}{1+\exp\left(-t/\tau_{Q}\right)}.$$

The function (31) is plotted in the middle graph of Figure 4. It is a sigmoid function describing a voltage transition from an IP ($\varphi =-V_{ex}$) to an FP (φ=0 or $V=V_{BD}$) corresponding to the damped oscillator transition curves in Figure 2a,b plotted as the orange color lines. The origin of the time axis is set at the time when $\varphi \left(t\right)$ reaches the midpoint of the path, i.e., $\varphi =-{(1/2)\cdot V}_{ex}$. At this point, from (27), we obtain the maximum value of $\dot{\varphi }$ as $V_{ex}/(4\tau_{Q})$ which is proportional to ${V_{ex}}^{2}$ (according to (6)) and gives the maximum “destabilization” energy (29). We point out that (31) is identical to a classical “instanton” solution with the potential energy given by $-A\left(\varphi \right)$ (negative of (28)) ([32], pp. 403–416), a highly localized function on the time axis treated as a particle. It is also noted that $\tau_{Q}$ is a half of the characteristic transition period as indicated in the middle graph of Figure 4. In this sense, $\tau_{Q}$ is regarded as the half lifetime of the instanton under a low R limit.

The AEP of the SPAD initially biased by a negative $V_{ex}$ is plotted as a dotted dark blue line showing local minima at φ=0 V and $\varphi =1.5\;V=(-V_{ex})$ in the top graph of Figure 4. Although symmetry breaking is introduced at the initial condition, φ=1.5 V, the response of the system is completely different from the IMPATT diode case. Because the initial condition at the second minimum point corresponds to a stable point of the Lienard equation, the output is fixed, as represented by a dotted straight dark blue line in the middle graph in Figure 4. This signifies the role of $V_{ex}$.

### 4.2. Capacitive Quenching Regime of SPADs

Next, we consider the opposite limit, R→∞, where the SPAD is capacitively quenched [23]. In this case, (7) leads to

(32) $$\ddot{\varphi }+\frac{1}{\tau_{Q}\cdot V_{ex}}\cdot \varphi \cdot \dot{\varphi }=0.$$

Note that (32) is equivalent to $\frac{d}{dt}\left(\dot{\varphi }+\frac{1}{\tau_{Q}\cdot V_{ex}}\cdot \frac{1}{2}\cdot \varphi^{2}\right)=0$, and therefore, $\dot{\varphi }+\frac{1}{\tau_{Q}\cdot V_{ex}}\cdot \frac{1}{2}\cdot \varphi^{2}=const.$ As shown in Section 2, in the capacitive quenching regime, the IP is $\left(\varphi ={-V}_{ex},\;\dot{\varphi }=0\right)$ and the FP is ($\varphi =V_{ex}$, $\dot{\varphi }=0)$. Therefore, the above equation for $\dot{\varphi }$ is obtained as

(33) $$\dot{\varphi }=-\frac{1}{2}\frac{1}{\tau_{Q}\cdot V_{ex}}\cdot \left(\varphi +V_{ex}\right)\cdot \left(\varphi -V_{ex}\right),$$

giving the above constant as $\frac{1}{2}\frac{V_{ex}}{\tau_{Q}}=\frac{1}{2}\frac{{V_{ex}}^{2}}{\tau^{\prime }}$, where we use (6). Then, the AEP can be obtained as

(34) $$A\left(\varphi \right)=\frac{1}{2}{\left(\dot{\varphi }\right)}^{2}=\frac{1}{8}\cdot \frac{1}{{\tau_{Q}}^{2}\cdot {V_{ex}}^{2}}\cdot {\left(\varphi +V_{ex}\right)}^{2}\cdot {\left(\varphi -V_{ex}\right)}^{2}.$$

Equation (34) is plotted as a blue line in the top graph of Figure 4. The IP and the FP are the two local minima, whereas the origin corresponding to $V=V_{BD}$ is the local maximum, indicating an instability with the destabilization energy of

(35) $$A\left(0\right)=\frac{1}{8}\cdot \frac{{V_{ex}}^{2}}{{\tau_{Q}}^{2}},$$

which is four times larger than that of the low-resistance regime given by (29) according to the scaled range of $\varphi \left(t\right).$ It is noted that the impact ionization process continues even after φ falls below $0\;(V=V_{BD})$, driven by the destabilization energy (35). This is attributed to the fact that the violation of the Lyapunov stability, i.e., $R>R_{L}$, leads to instability or a tendency of V to run away from $V_{BD}$. Although the available energy (34) holds symmetry with respect to the origin (the vertical axis), the minimum energy state deviates from the origin (center of the gravity of the profile) as $\varphi =\pm V_{ex}$. Thus, the capacitive quenching mode is regarded as a symmetry-broken phase [33]. It is also noted that at the FP, the number of charges in the MR is zero or the system is in a complete CE state. This is a high contrast to the low R case in which infinite charges are generated at the FP.

Returning to (33), it is also a type of logistic equation with an exact solution as follows [23].

(36) $$\varphi \left(t\right)=V_{ex}\cdot \frac{1-\exp\left(-t/\tau_{Q}\right)}{1+\exp\left(-t/\tau_{Q}\right)}=V_{ex}\cdot \tanh\left(t/\left({2\tau }_{Q}\right)\right).$$

Here, the origin of the time axis is set at which φ=0 or $V=V_{BD}.$ At this point, $\dot{\varphi }$ assumes the maximum value of $\frac{1}{2}\frac{V_{ex}}{\tau_{Q}}=\frac{1}{2}\frac{{V_{ex}}^{2}}{\tau^{\prime }}$, twice the maximum value in the low-resistance (IMPATT) regime. It is also scaled with ${V_{ex}}^{2}$. As with (31), this solution is also identical to a classical “instanton” solution with the negative of (34) as the potential energy ([32], pp. 403–416). And $\tau_{Q}$ also corresponds to its half lifetime as indicated in the middle graph of Figure 4 with blue arrows.

Thus, the above analysis points to the high similarity of AEP, $A\left(\varphi \right)$, to the free energy in the GL theory of phase transition; not only the forms of the functions, the behavior that exhibits a transition from a symmetric character to a symmetry-broken one induced by the variation in the value of the QR. It is also noted that φ resembles an order parameter in the Ginzburg–Landau theory ([26], pp. 110–147) specifying the energy minima; φ=0 for the low R case (symmetric AEP); $\varphi =\pm V_{ex}$ for the limit, R→∞, (symmetry-broken AEP).

### 4.3. Resistive Quenching Regime of SPADs

A successful resistive quenching path is plotted as dotted yellowish lines in Figure 2a,b. The corresponding available energy, $A\left(\varphi \right)$, is plotted in the top graph of Figure 5a as a solid yellow line by calculating $\frac{1}{2}{\left(\dot{\varphi }\right)}^{2}$ of the data used in Figure 2. In the bottom, the corresponding time-dependent signal, $\varphi \left(t\right),$ is plotted. In the early period up to TP designated by a blue arrow (designated as $t_{TP}$ on the time axis in the bottom figure), $A\left(\varphi \right)$ follows close to that of the double well profile of the CQ (34) (dotted light blue line). This is the (depletion) discharge phase closely represented by a classical instanton term (34) observed as a sharp increase with an effective rise time of ~${2\tau }_{Q}$. Then, the system snaps back to the IP ($\varphi =-V_{ex})$ by recharging the MR (or discharging the stored electrons on C). Although the path from TP to IP appears as being nearly flat along the horizontal axis in Figure 5a, it is not the case as shown in the magnified plot of Figure 5b. This period is then regarded as another phase since the path in the phase space and the corresponding $A\left(\varphi \right)$ are represented by a different function as follows. The analytical expression of the phase space is obtained by setting $\tau_{Q}\to \infty$ in (7) and by integrating once,

(37) $$\dot{\varphi }+\frac{1}{R\cdot C}\cdot \varphi =Const.,$$

giving a linear line consistent with the one plotted in Figure 3b. Taking account of the fact that, at the end of the recharge phase, the system returns to the IP ($\varphi =-V_{ex})$ with the minimum energy (=0), the profile of $A\left(\varphi \right)$ is obtained as

(38) $$A\left(\varphi \right)=\frac{1}{2}{\left(\dot{\varphi }\right)}^{2}=\frac{1}{2}\frac{1}{{\left(RC\right)}^{2}}\cdot {\left(\varphi +V_{ex}\right)}^{2}.$$

Equation (38) is plotted as a dotted purple line in Figure 5b. After the recharge phase takes place, $n\left(t\right)$ drops down “below” unity. At this point, the CE condition is introduced as indicated by a red arrow. The corresponding points are also indicated in Figure 5a as CE and $t_{CE}$. This is the most important condition to be met, without which the system always goes back to the spiral stable phase due to avalanche multiplication of “fractional charge” [24]. It is also emphasized that CE should take place before φ reaches the vertical axis, φ [23]. Since at the origin φ=0, $A\left(\varphi \right)$ is not a local minimum due to the application of $V_{ex}$; thus, these phases are also regarded as symmetry-broken ones [33].

## 5. Instanton Model

### 5.1. General Remark

In this section, the classical solutions of the LE (7) are extended to a quantum mechanical regime describing instantons, solitary wave functions highly localized in time, by a path integral [27], ([32], pp. 31–73). The voltage function φ(t) is now regarded as an observable of a quantum system and a quantum state located at a voltage φ at time t is represented as $\left|\varphi \right.,\;\left.t\right\rangle .$ Then, a probability amplitude of the system propagates from an initial state, $\left|\varphi =V_{i}\right.,\;\left.t=0\right\rangle ,$ to a final state $\left|\varphi =V_{f}\right.,\;\left.t=T\right\rangle$, or a Green’s function $G\left(V_{f},\;T/2,{\;V}_{i},\;-T/2\right)$ is described by a path integral as follows ([27], pp. 210–219), ([32], pp. 31–73).

(39) $$G\left(V_{f},\;T,V_{i},\;0\right)\equiv \left\langle V_{f},T|V_{i},\;0\right\rangle =\int D\left[\varphi \left(t\right)\right]\cdot e^{\frac{iS\left[\varphi \left(t\right)\right]}{\hbar }},$$

where $D\left[\varphi \left(t\right)\right]$ describes an integration over all possible paths of $\varphi \left(t\right)$ derived from a given Lagrangian or a Hamiltonian and $S\left[\varphi \left(t\right)\right]$ is an action integral of the entire time range defined as

(40) $$S\left[\varphi \left(t\right)\right]\equiv \int_{0}^{T}L(\varphi ,\dot{\varphi })dt=\int_{0}^{T}\left\{\frac{1}{2}{\left(\dot{\varphi }\right)}^{2}-V\left(\varphi ,\dot{\varphi }\right)\right\}dt,$$

where $L(\varphi ,\dot{\varphi })$ and $V\left(\varphi ,\dot{\varphi }\right)$ are the Lagrangian and the potential energy of the system. To compute (39), the stationary phase approximation in which dominant contribution is due to the paths close to the classical solution, $\varphi_{C}$, is assumed [27]. Expanding $S\left[\varphi \left(t\right)\right]$ around $\varphi_{C}$ as

(41) $$S\left[\varphi \left(t\right)\right]=S_{C}+\frac{1}{2!}S^{\prime \prime }\left[\varphi_{C}\right]\cdot {\left(\varphi -\varphi_{C}\right)}^{2}+\cdots ,$$

where $S_{C}$ is an action of the classical solution ($\equiv S\left[\varphi_{C}\left(t\right)\right]$). The first-order term disappears, i.e., $S^{\prime }\left[\varphi_{C}\right]=0,$ because of the assumed stationarity at the classical solution leading to the Euler–Lagrange equation (14) ([27], pp. 210–219). Then, keeping only up to the second order, (39) leads to

(42) $$G\left(V_{f},\;T,V_{i},\;0\right)=e^{\frac{iS_{C}}{\hbar }}\cdot N\cdot {\left[det^{\prime }\left\{i\cdot \left(-\frac{d^{2}}{dt^{2}}-V^{\prime \prime }\left(\varphi_{C}\right)\right)\right\}\right]}^{-1/2},$$

where $det^{\prime }\left\{i\cdot \left(-d^{2}/dt^{2}-V^{\prime \prime }\left(\varphi_{C}\right)\right)\right\}$ describes the determinant of the operator i·(${-d}^{2}/dt^{2}-V^{\prime \prime }\left(\varphi_{C}\right)$) with positive eigenvalues. For zero-valued eigenvalues (called “zero-mode”), a separate calculation is necessary and the normalization coefficient, N, contains these factors [34,35]. Thus, the form of (42) is the classical solution $e^{\frac{iS_{C}}{\hbar }}$, modified by quantum fluctuations described by the second and the third terms ([27], pp. 210–219).

To calculate (42), we need $S_{C}$, the integrand of which is the Lagrangian given as (40). An explicit form of $V\left(\varphi ,\dot{\varphi }\right)$, effective potential energy, is not given so far. It is derived by utilizing the total energy conservation derived as (17). Let $E_{0}$ be the total energy, then V is obtained by simply subtracting the kinetic energy term $A\left(\varphi \right)$ as

(43) $$V\left(\varphi \right)=E_{0}-A\left(\varphi \right).$$

Thus, $V\left(\varphi \right)$ is obtained as the negative of $A\left(\varphi \right)$ derived in Section 4 with a proper choice of a reference level. Then, the solutions or the trajectories of the LE (7) are represented by one-particle dynamics moving from the IP to the FP along the potential curves. These are the classical “instantons” solutions localized in a narrow region of time axis. As for the quantum corrections, they are obtained as integrals of the second-order terms after the expansion of (41). This involves eigenvalue problems as resolved by the determinant term in (42). Fortunately, in the case where an exactly solved classical instanton such as (36) is available, complete solutions of (42) are calculated in a form of a Euclidian kernel ([32], pp. 403–416), [34,35]. In Appendix B, the Hamiltonian and the eigenvalue equation of the capacitive quenching solved with a Feynman kernel are equivalent to those of the above. Thus, to apply these results, we convert $e^{\frac{-S_{C}}{\hbar }}\to e^{\frac{iS_{C}}{\hbar }}$ and multiply a phase factor with a unit amplitude. Therefore, as far as the absolute value of the determinant is concerned, the quantum correction parts are the same between the two kernels. Utilizing this procedure, an expression for the avalanche triggering probability is derived in Appendix C. In the following, we will make use of this result. A general form of the absolute value of the Green’s function is as follows.

(44) $${\left|G\left(V_{f},\;T,V_{i},\;0\right)\right|}^{2}=\frac{1/\tau_{Q}}{sinh(T/\tau_{Q})}\cdot S_{C}\cdot 1/{\tau_{Q}}^{2}\times \left|Const.\right|,$$

where $\left|e^{\frac{iS_{C}}{\hbar }}\right|=1$ is used. The first term on the right-hand side describes a probability density that the system remains at the IP, at the top of the flipped well, with an unstable status. In a long time limit, ($T\gg \tau_{Q})$, the term approaches as $\frac{1/\tau_{Q}}{sinh(T/\tau_{Q})}\to \frac{1}{\tau_{Q}}\cdot \exp\left(-\frac{T}{\tau_{Q}}\right)=\frac{V_{ex}}{\tau^{\prime }}\cdot \exp\left(-\frac{T{\cdot V}_{ex}}{\tau^{\prime }}\right)$. Such an exponential probability density distribution function of the transition probability is consistent with the reported experimental results on the avalanche triggering probability [5,17,36]. The second term, $S_{C},$ the action of the classical solution defined above, arises from the solution of the zero-valued eigenvalue problem [34,35,37]. Since this is the most dominant term, we will focus on the calculation of the term in the following. The third term also arises from the normalization procedure of the zero-mode solution. Because of the $V_{ex}$ dependence of $\tau_{Q}$, its effect should be included in the final expression. Finally, the fourth term (represented as a positive constant) includes contributions from the other eigenvalue solutions and undetermined integration parameter. Detailed values of these terms do not affect the main conclusions of the present study and will not be discussed further [34,35,36].

### 5.2. Capacitive Quenching Regime

As a typical example of an instanton model, we start with the capacitive quenching (R→∞) regime. From (35) and (43), and by setting $E_{0}=0$, the potential profile is obtained as

(45) $$V\left(\varphi \right)=-\frac{1}{8}\cdot \frac{1}{{\tau_{Q}}^{2}\cdot {V_{ex}}^{2}}\cdot {\left(\varphi +V_{ex}\right)}^{2}\cdot {\left(\varphi -V_{ex}\right)}^{2}.$$

The profile of $V\left(\varphi \right)$ is a vertical flip of $A\left(\varphi \right)$ plotted as a blue line in the bottom graph of Figure 4. It describes a potential profile for a particle set at IP at the top of the left-side potential hill to descend with increasing kinetic energy and, after passing the bottom, to reach the top of the other hill. From (13) and (45), the Lagrangian of the system is then expressed as

(46) $$L\left(\varphi \right)={\left(\dot{\varphi }\right)}^{2}=-2V\left(\varphi \right)=\frac{1}{4}\cdot \frac{1}{{\tau_{Q}}^{2}\cdot {V_{ex}}^{2}}\cdot {\left(\varphi +V_{ex}\right)}^{2}\cdot {\left(\varphi -V_{ex}\right)}^{2}.$$

Now, from (40) and (46), we obtain the classical solution of action as

(47) $$S_{C}=\int_{0}^{T}L(\varphi ,\dot{\varphi })dt=\int_{0}^{T}{\left(\dot{\varphi }\right)}^{2}dt=\int_{0}^{T}\dot{\varphi }\cdot d\varphi =\int_{-V_{ex}}^{V_{ex}}\sqrt{\left|-2V\left(\varphi \right)\right|}\cdot d\varphi =\frac{2}{3}\cdot \frac{{V_{ex}}^{2}}{\tau_{Q}}=\frac{2}{3}\cdot \frac{{V_{ex}}^{3}}{\tau^{\prime }}.$$

where $\tau^{\prime }$ is a specific ionization ratio defined in (6). Thus, $S_{C}\propto {V_{ex}}^{3}$. Taking account of (44), the probability of transition is described as

(48) $${\left|G\left(V_{f},\;T,V_{i},\;0\right)\right|}^{2}=\frac{V_{ex}/\tau^{\prime }}{sinh(T/\tau_{Q})}\cdot \frac{{V_{ex}}^{3}}{\tau^{\prime }}\cdot {V_{ex}}^{2}/{\tau^{\prime }}^{2}\times \left|Const.\right|,$$

which is proportional to ${V_{ex}}^{6}$. This means that the larger the excess bias, $V_{ex}$, the higher the transition probability or the probability of successful capacitive quenching. The increasing factor is not linear but exponential. Both tend to agree with practical experiences. Also, because $S_{C}\propto {V_{ex}}^{3}$, a larger $V_{ex}$ leads to an oscillation with higher frequency in the first classical term, $e^{\frac{iS_{C}}{\hbar }}$, increasing canceling of out-of-phase components due to the increased interference effect ([27], pp. 210–219). This means the term is eventually dominated by the classical term with increasing $V_{ex}$.

### 5.3. Low-Resistance (IMPATT Diode) Regime

In a similar way of deriving (45), using (29), the effective potential profile of the low-resistance (IMPATT diode operation) regime is obtained as

(49) $$V\left(\varphi \right)=-\frac{1}{2}\cdot \frac{1}{{\tau_{Q}}^{2}\cdot {V_{ex}}^{2}}\cdot \varphi^{2}\cdot {\left(\varphi +V_{ex}\right)}^{2},$$

where $E_{0}=0$. The profile of $V\left(\varphi \right)$ is also a vertical flip of $A\left(\varphi \right)$ plotted as an orange line in the bottom graph of Figure 4. The corresponding one-particle dynamics is similar, too; a particle set at IP at the top of the left-side potential hill to descend and to climb up to the top of the other hill. From (13) and (49), the Lagrangian is calculated as

(50) $$L\left(\varphi \right)={\left(\dot{\varphi }\right)}^{2}=-2V\left(\varphi \right)=\frac{1}{{\tau_{Q}}^{2}\cdot {V_{ex}}^{2}}\cdot \varphi^{2}\cdot {\left(\varphi +V_{ex}\right)}^{2}.$$

Taking account of the integration region of the voltage; $-V_{ex}\leq \varphi \leq 0$, (40) and (50) leads to the classical solution of action as

(51) $$S_{C}=\int_{0}^{T}L(\varphi ,\dot{\varphi })dt=\int_{0}^{T}{\left(\dot{\varphi }\right)}^{2}dt=\int_{0}^{T}\dot{\varphi }\cdot d\varphi =\int_{-V_{ex}}^{0}\sqrt{\left|-2V\left(\varphi \right)\right|}\cdot d\varphi =\frac{1}{6}\cdot \frac{{V_{ex}}^{2}}{\tau_{Q}}=\frac{1}{6}\cdot \frac{{V_{ex}}^{3}}{\tau^{\prime }}.$$

where $\tau^{\prime }$ is a specific ionization ratio defined in (6). Thus, we confirm $S_{C}\propto {V_{ex}}^{3}$, again. The difference in magnitude (coefficient) is due solely to reduction in the integration region, ${2V}_{ex}\to V_{ex}$.

### 5.4. Recharge Phase of a Resistive Quenching Regime

As discussed in Section 4.3, after reaching a TP and realizing CE, the system enters into a recharge phase where the profile follows the form (38). Along the trajectory, the system moves in the opposite direction on the voltage axis to the instanton or the discharge pulse described in 5.2 with negative momentum ($\dot{\varphi }<0$). In this sense, the transition may be called an anti-instanton ([27], pp. 457–466). Denoting the time and the voltage of the system at TP as $T_{TP}$ and $V_{TP}$, a main contributing factor is an action calculated similarly to above as follows.

(52) $$S_{C}=\int_{T_{TP}}^{T}L(\varphi ,\dot{\varphi })dt=\int_{T_{TP}}^{T}{\left(\dot{\varphi }\right)}^{2}dt=\int_{V_{TP}}^{{-V}_{ex}}\sqrt{\left|-2V\left(\varphi \right)\right|}\cdot d\varphi =-\frac{1}{2RC}\cdot {\left(V_{TP}+V_{ex}\right)}^{2}.$$

We note that the negative sign indicates the character of the anti-particle. Such a viewpoint of a GM-pulse as a pair of an instanton and an anti-instanton is described in the bottom part of Figure 5a. We emphasize that only when this pair is formed, which is possible only when the CE condition is met, a successful quenching is realized.

## 6. Discussion

### 6.1. Signatures of the Phase Transitions

The analyses so far clearly show that completely different modes of the carrier dynamics in an avalanche diode could arise from a single nonlinear LE (7) by parametric variation in the series-resistance or the QR value. Thus, the phenomenon would be regarded as a phase transition governed by the QR value. In Figure 6a, the value of $\dot{\varphi }$ at $\varphi \;\left(t\right)$ = 0 calculated with the same parameters listed in Table 1 is plotted as a function of R with a blue-colored plot referenced to the left vertical axis. It is found that there is a threshold or a transition point at which $\dot{\varphi }$ starts to exhibits positive values from the constant zero. The threshold value is interpolated to be $R_{th}$ (=$\tau_{Q}/\left(4C\right)=550\;\Omega$ [25]), the bifurcation point at which the character of the fixed point $\left(\varphi =0,\dot{\varphi }=0\right)$ transforms from a stable node to a stable spiral sink. Thus, the bifurcation point is identified as a phase transition point of the carrier dynamics or the operational mode. From this point, an “instability” at $V_{BD\;}\;(\varphi =0$) emerges, leading to symmetry breaking in A(φ). The transition is continuous but the order is not undetermined at this stage.

There are two more points at which the system’s stability dramatically changes as discussed in Section 2 and Section 3; $R_{Q}$ (9) is a value beyond which CE is in effect and therefore a successful quenching is possible. For a successful quenching, $R>R_{Q}$ must be satisfied; $R_{L}$ (26), below which the system is sure to spiral into the steady state $\left(\varphi =0,\dot{\varphi }=0\right)$ even though an overshoot beyond the BV (φ=0) is possible. These points are also designated in Figure 6a. Thus, the phase of an SPAD operation is classified into the following regions depending on the value of R.

(i) Overdamped (OD) region with $0<R<R_{th}$; any initially applied EV introducing symmetry breaking into the AEP is brought back to 0 within $\sim {2\tau }_{Q}$ without any overshoot. The BV point is stable in the end. Thus, the region is termed as quasi-symmetric. The pulse is not quenchable.
(ii) Spiral sink (SS) region with $R_{th}\leq R\leq R_{L}(26)$; due to the first inequality, the BV point is unstable and true symmetry breaking emerges (Figure 6a). Due to the second inequality (Lyapunov stability condition), φ is surely brought back to 0 in the end, recovering the symmetry in the AEP (the pulse is not quenchable).
(iii) Fluctuation/Oscillation (FO) with $R_{L}\leq R\leq R_{Q}\left(9\right).$ Because the Lyapunov stability is violated (first inequality), φ is not necessarily brought back to 0. The pulse is not quenchable due to the second inequality. The BV point is intrinsically unstable and its symmetry is regarded as completely broken.
(iv) A region where a successful quenching (SQ) is possible with $R_{Q}<R$. The GM-pulse is represented as an instanton–anti-instanton pair.

The above descriptions for the four phases are summarized in Table 2.

Since all the values of $R_{th}$, $R_{L}$, and $R_{Q}$ are described as functions of $\tau_{Q}$ which are, in turn, inversely proportional to $V_{ex}$, the phase of the avalanche device mode is mapped on a two-dimensional $R-V_{ex}$ space (R>0, $V_{ex}\ll V_{BD})$. An example of a phase map calculated for an SPAD with the same parameters as those of Figure 3 is constructed in Figure 6b.

### 6.2. The Role of the Carrier Extinction (CE) and the Meaning of $R_{Q}$

In Figure 6a, the voltage margin to the BV at which CE takes place, $\varphi_{CE}\equiv V_{BD}-V_{CE}(t)$, numerically calculated as a function of QR, is plotted with a green line plot referenced to the right-side vertical axis. Starting from $R\sim R_{Q}$, $\varphi_{CE}$ sharply rises, exhibiting a phase transition character. $\varphi_{CE}$ increases with the increase in QR in accordance with the fact that a higher QR leads to a higher probability of successful quenching [3]. This phase transition is interpreted as a transition of the stability character of the breakdown voltage point from a spiral sink to the unstable point as discussed in Section 4.3. Thus, CE gives the idling point ($\varphi =-V_{ex})$ a local minimum character in the AEP with a broken-symmetry and enables the system to recover the idling state, i.e., a successful quenching.

The physical meaning of $R_{Q}$ is elucidated by considering the quantum uncertainty principle between the single charging energy and the time as follows. The energy uncertainty, $∆E_{QM}$, due to a single charge on a capacitor with a capacitance value C, is $∆E_{QM}=q^{2}/\left(2C\right).$ Then, the quantum mechanical time uncertainty, $∆t_{QM}$, is obtained as

(53) $$∆t_{QM}\sim \frac{h}{2}\cdot \frac{2C}{q^{2}}=R_{QM}\cdot C,$$

where h is the Planck’s constant and $R_{QM}$ = $h/\left(q^{2}\right)\sim 26\;k\Omega$ is the quantum resistance or the inverse of the Landaure quantum conductance of single charge [38,39]. It is emphasized that the value $2R_{QM}\sim 52\;k\Omega$ is nearly identical to the rule of thumb set by [3]. Thus, the required condition by the CE would be restated as follows; the time constant of the recharging should be longer than the quantum mechanical time uncertainty corresponding to single-charge charging. This means that the final single charge on the storage capacitance should not be recharged by the quantum mechanically allowed $R_{QM}C$-charging process requiring a larger value of QR (${\geq 2R}_{QM})$, which provides sufficient negative feedback effect to ensure the quenching.

### 6.3. Construction of a Photon Counting State

In the following, utilizing the instanton–anti-instanton pictures discussed in Section 5, descriptions for a series of successfully quenched GM-pulses are obtained. At a time long after the initial condition is set, we suppose that an SPAD under a resistively quenchable condition is in a quantum mechanical state with the output node voltage $\varphi ={-V}_{ex}$ after the k-th GM-pulse quenched and is denoted as $\left|\left.{-V}_{ex},\;k\right\rangle \right.$. We also assume that photons arrive at a rate much smaller than the inverse of the recharge phase time constant, $1/\tau_{RC}$, following a Poisson distribution. A probabilistic event of a single-photon absorption by the system in this state is described by a photon absorption operator, $\hat{a}$, and a quantum efficiency of the absorption, η, as follows.

(54) $$\hat{a}\left|\left.{-V}_{ex},\;k\right\rangle \right.=\sqrt{\eta }\cdot \left|\left.{-V}_{ex},\;k\right\rangle \right..$$

Next, denoting an operator that generates one instanton $b^{†}$ transferring the system from $\left|\left.{-V}_{ex},\;k\right\rangle \right.$ to $\left|\left.V_{TP},\;k\right\rangle \right.$ with a Green’s function $G\left(V_{TP},\;T/2,{-V}_{ex},\;-T/2\right)\equiv K_{I}$, an event of a single instanton generation due to a single carrier injection is described as

(55) $$b^{†}\left|\left.{-V}_{ex},\;k\right\rangle \right.=K_{I}\cdot \left|\left.V_{TP},\;k\right\rangle \right..$$

Similarly, after passing through TP, an anti-instanton is generated. By denoting a CE operator as $\hat{c}$, the probability amplitude of the occurrence of the CE as $\sqrt{\kappa },$ anti-instanton generation operator as $d^{†}$ and the amplitude of a Green’s function $G\left({-V}_{ex},\;T/2,V_{TP},\;-T/2\right)$ as $K_{AI}$, the probabilistic recharge process is described as

(56) $${\hat{c}d}^{†}\left|\left.V_{TP},\;k\right\rangle \right.=\sqrt{\kappa }\cdot K_{AI}\cdot \left|\left.-V_{ex},\;k+1\right\rangle \right..$$

In order to successfully quench the GM-pulse during the recharge phase, a probabilistic event of CE should be realized as above. Redefining the operators, $d^{\prime †}\equiv \hat{c}d^{†}$ and $b^{\prime †}\equiv b^{†}\hat{a}$, as complete generation processes of an anti-instanton and an instanton, respectively, a single-photon detection event followed by a single successfully quenched GM-pulse is described as

(57) $$d^{\prime †}b^{\prime †}\left|\left.{-V}_{ex},\;k\right\rangle \right.=K_{AI}\cdot \sqrt{\kappa }\cdot K_{I}\cdot \sqrt{\eta }\cdot \left|-\left.V_{ex},\;k+1\right\rangle \right..$$

We note that the voltage is unchanged (in the idling state) as $\varphi ={-V}_{ex}$ after the event while the number index of k is increased by 1. Then, squaring the coefficient of the right-hand side of (57), a photon detection efficiency, $P_{PDE}$, is obtained as

(58) $$P_{PDE}=\eta \cdot {\left|K_{AI}\right|}^{2}\cdot \kappa \cdot {\left|K_{I}\right|}^{2}.$$

Thus, PDE is modified by the probability associated with the intrinsic carrier dynamics of the GM-pulse, i.e., instanton (${\left|K_{I}\right|}^{2}$), CE (κ), and anti-instanton (${\left|K_{AI}\right|}^{2}$), in addition to a common term (η) which would include material’s absorption coefficient, and geometrical and or structural factors.

Then, the probability of k-time successful photon detections (GM-pulse generation) out of n arrived photons, $P_{n}(k)$, is described as a binominal distribution as

(59) Pn(k)=Ckn·1−PPDEn−k·PPDEk.

Combining (54)–(58), and based on (59), a quantum mechanical wave function describing the entire photon counting process out of n photons is constructed as

(60) $${\left|\left.\Psi \right\rangle \right.}_{\;n}=A\cdot {\left\{{\left(1-P_{PDE}\right)}^{\frac{1}{2}}+\exp\left(i\theta \right)\cdot d^{\prime †}\cdot b^{\prime †}\right\}}^{n}\left|-\left.V_{ex},\;0\right\rangle \right.,$$

where A is a normalization constant, and θ is a possible phase factor associated with a quantum mechanical process. With n=1, (60) describes a superposition of a successfully GM-pulse-quenched state after one photon detection, $\left|-\left.V_{ex},\;1\right\rangle ,\right.$ and a failure state, $\left|-\left.V_{ex},\;0\right\rangle \right.$. Expanding the right-hand side, a coefficient containing the square of the k-th order term of ($d^{\prime †}\cdot b^{\prime †})$ is proportional to $P_{n}(k)$.

The second term in the curly parenthesis of (60) describes a steady state of instanton–anti-instanton pairs recovering to the initial bias condition or a series of successful quenching processes. If only an instanton is generated and no anti-instanton is created, then the system remains in a state ~$\left|\left.V_{TP},\;k\right\rangle \right.$ failing to recover the idling state. Although it might sound obvious from an experimental point of view, considering theoretical assumptions, its implications are significant. Firstly, a common assumption of the instanton theory is that a period between an instanton and anti-instanton should be sufficiently long that there is no correlation between the adjacent particles ([27], pp. 457–466), [37]. The resulting waveform is a series of rectangular pulses exhibiting a random telegraph noise signal ([27], pp. 457–466), [37]. In addition, the average value of the signal (expectation value) is 0, recovering the symmetry. The situation is illustrated in Figure 7b. One important aspect of this result is that the above model is capable of producing the phenomenon of the long-time studied micro-plasma [17,40] only by the random transitions between the bistable potential wells. The model’s relation and/or combination with common models based on traps [41] would be worthwhile for investigation. This is a high contrast with the present case (Figure 7a), where each time an instanton–anti-instanton pair is generated and the system predominantly sits in the idling (one of the minima of the double well) state after many GM-pulses are quenched. In this sense, we note similarity with superconductivity. The algebraic structure where a particle–anti particle pair is involved is analogous to the Bardeen–Cooper–Schriefer (BCS) wave function of superconductivity ([26], p. 50). By forming the Cooper pairs, the system takes one of the two lowest energy states in a symmetry-broken energy profile (double well). While electron–phonon interaction is responsible for forming the Cooper pair in superconductivity, CE makes the coupling or pairing of instanton–anti-instanton possible by effectively making $\tau_{Q}$ infinite.

In the above treatment, the voltage parameter is apparently promoted to a parameter describing the quantum state φ without mathematical justification. However, the quantization conditions of φ in the Lienard equation derived in Appendix A are considered to be a strong support for such treatment in the present case.

### 6.4. Analogy with Superconductivity

In the course of analyses so far, we find that the collective carrier dynamics of an avalanche diode is divided into distinguishing modes or phases by the value of the QR. The solution of the LE, φ(t), defined in the “time” domain forms an AEP, $A\left(\varphi \right)$, giving the phase transformation from the symmetric phase in the low R limit to the symmetry-broken one in the large R limit. We have found the situation is analogous to the commonly discussed phase transition of “spatially” defined microstructures. The existence of such an analogy was pointed out a long time ago with a Fokker–Planck equation as a starting point [42]. Up to the present section, wherever noticed, we point out the similarity of the present case to the phenomenological descriptions in superconductivity. The relevant items are summarized in Table 3. Two notes should be made here. First, we recognize the caution that a fundamental difference between time-domain and space-domain phenomena clearly exists [43]. In the former including the present case, the system is in a non-equilibrium condition, while in the latter case, the system in equilibrium transforms its phase. The analogies of the items in the table are therefore in large part considered as formal ones—analytical and algebraic structures of the equations. Secondly, as described in Appendix A, the classical voltage parameter, φ(t), in the Lienard equation is quantized as a field. This is because the constructed Hamiltonian involves all the relevant energies including the dissipative ones and constitutes a conservative force field. This result appears to support mathematical consistency and the physical origin of pursuing the present analogies.

## 7. Conclusions

A phase transition model for the carrier dynamics in an SPAD is developed from the LE (7). With the LE, a Lagrangian (13) and a Hamiltonian (16) giving the LE by the Euler–Lagrange equation and by the Hamilton equation, respectively, are identified. From the Hamiltonian, energy conservation is also confirmed. A Lyapunov stability condition (26) is found to give an interfacial region between the quenchable and non-quenchable regions. An AEP is derived from the Lagrangian, and operational modes of a GM-operated avalanche device are classified into four phases represented by the AEP as a function of the QR value, R (Figure 6b, Table 2). With no EV (denoted as $V_{ex}$), the AEP phase is symmetric, corresponding to a DC-biased steady state (30). Application of a finite $V_{ex}$, GM-operation, is found to break the symmetry at the initial condition as indicated by the occurrence of a double well corresponding to “instability” near BV (29). In a low R (R→0) limit, the system is regarded as being in a quasi-symmetric phase corresponding to the operational mode of IMPATT devices. “Intrinsic” symmetry breaking is found to start when $R>R_{th}$, at BV (Figure 6a). The energy at BV is no longer a local minimum. Ultimately, in a R→∞ limit, the AEP exhibits a complete double well (34). A GM-pulse under a successful quenching condition is identified as a round-trip-transition between the local minima of the AEP represented as the double well (Figure 5). The entire phase diagram is constructed on a 2D $R-V_{ex}$ plane (Figure 6b). Then, using the identified Lagrangian (Hamiltonian) and the classical solution of the LE (31), (36), a quantum mechanical “instanton” model is developed by a Feynman path integral scheme (39). Green’s functions arising from transitions between local minima of the available energy’s double well are derived (44), (47), (51), (52). In particular, the avalanche discharge period and the recharge period during quenching are, respectively, identified as “instanton” and “anti-instanton” and a successfully quenched GM-pulse is found to be described as a pair of an instanton and an anti-instanton connected by a CE event (57) (Figure 5). A wave function describing a photon counting process is constructed as successive quenching pulses comprising a product of instanton–anti-instanton pairs (60). Finally, analogs of the SPAD carrier dynamics to the phenomenology of superconductivity are discussed (Table 3).

## Figures and Tables

**Figure 1:**
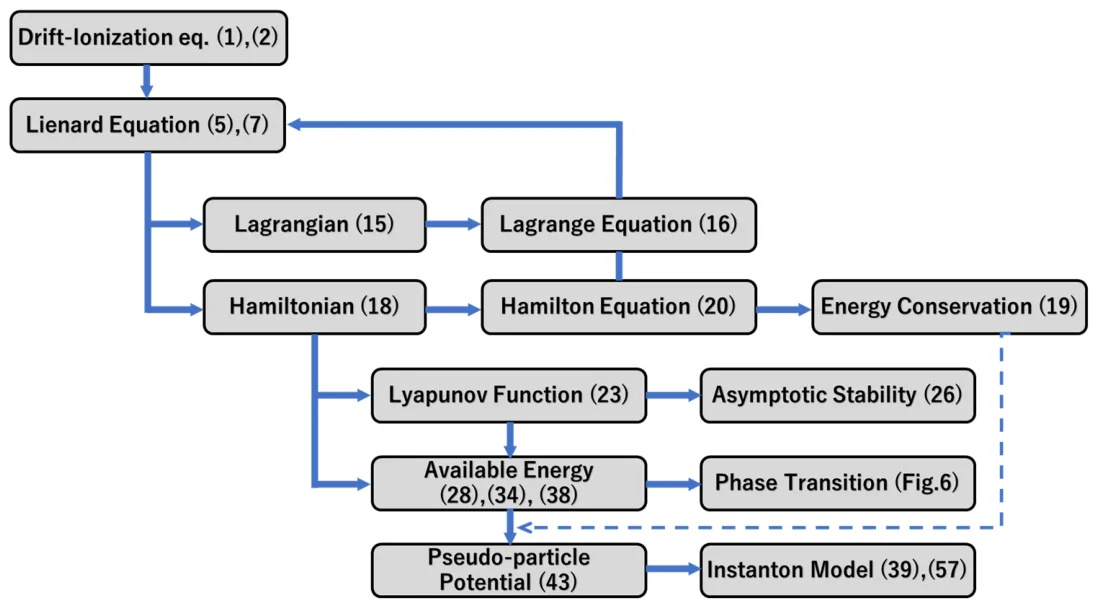
Flow of the analysis. The numbers in the parentheses are those of the corresponding equations in the body of the text. In the box of the phase transition, the corresponding number of the figure is given.

**Figure 2:**
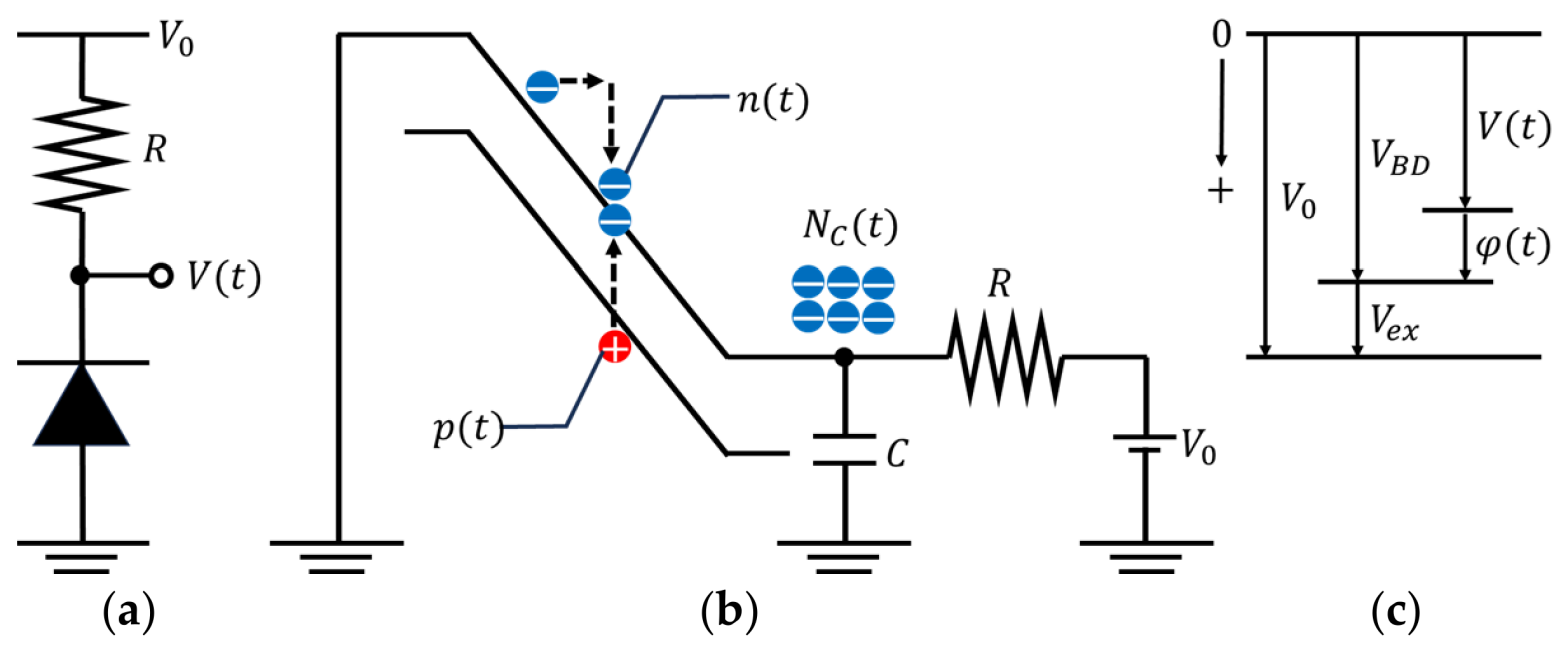
(**a**) A circuit diagram of an SPAD specifying a voltage source $V_{0}$, a QR, R and the time-dependent output voltage V(t). (**b**) A band diagram equivalent to diagram (**a**). (**c**) Definitions and relations of relevant voltage values. Zero voltage level is represented as a line at the top horizontal line. A positive voltage is represented by an arrow pointing downward as indicated on the left side.

**Figure 3:**
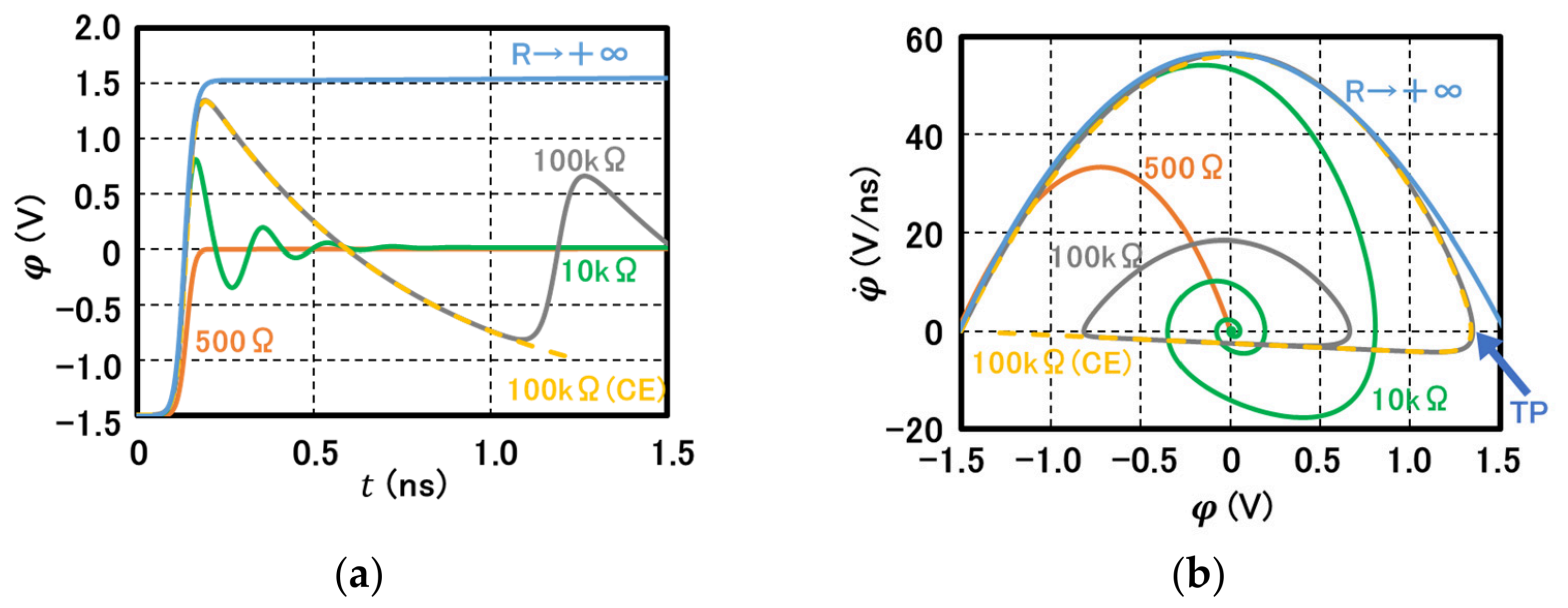
Numerical solutions of LE (7) for different QR values. (**a**) $\varphi \left(t\right)$ vs. time; (**b**) phase-space trajectories ($\dot{\varphi }\left(t\right)$ vs. $\varphi \left(t\right)$) calculated for the same set of QR values as plotted in (**a**). Dotted yellow lines are results under CE condition. TP is a turning point at which the sign of $\dot{\varphi }$ changes from positive to negative.

**Figure 4:**
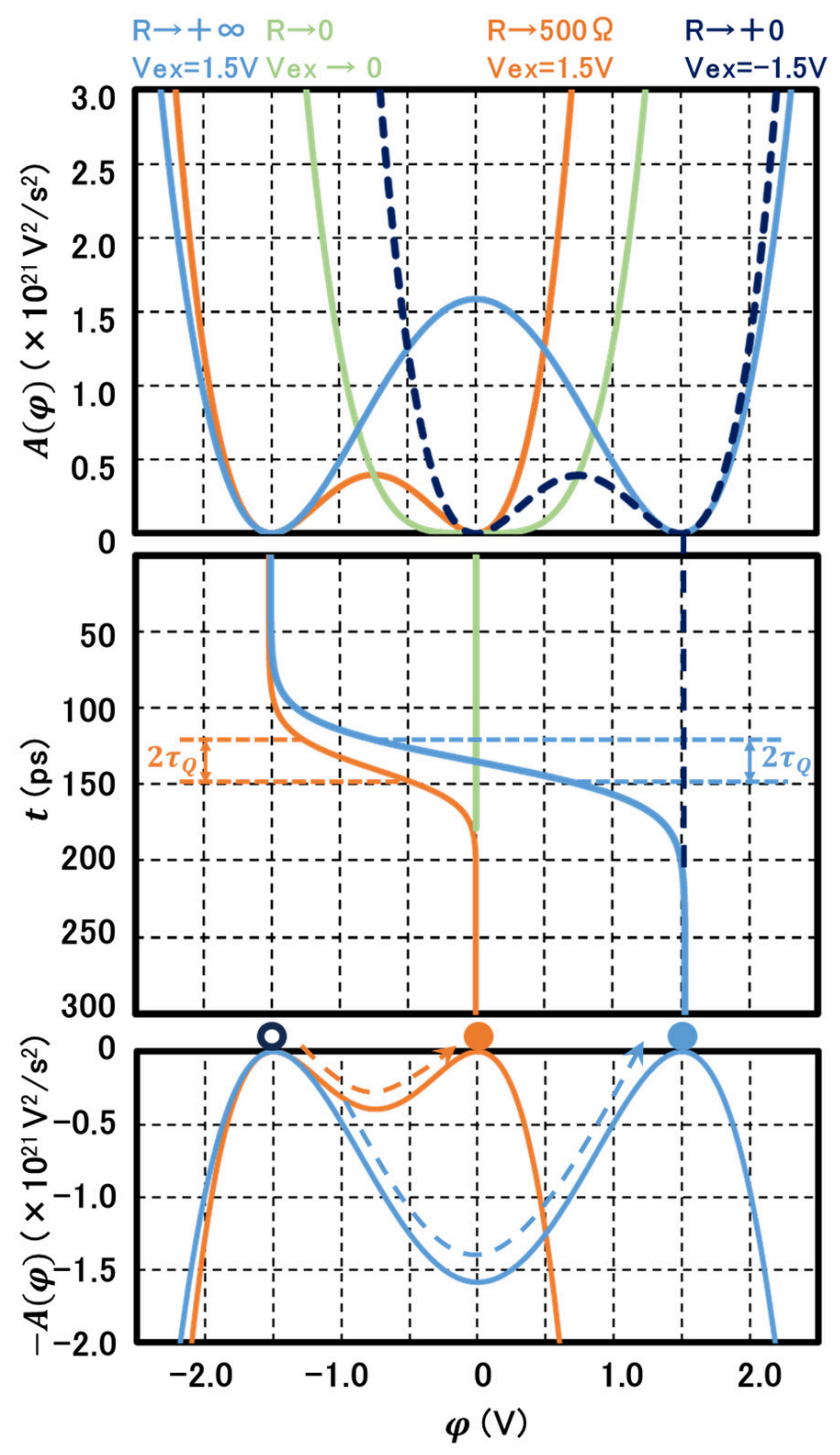
Profiles of available energy as functions of φ (**top**), voltage outputs φ as functions of time (**middle**), and profiles of instanton potentials with illustrations of one-particle movement (**bottom**) calculated for different values of QR as denoted by insertions with the same-colored letters as the corresponding plots. Physical parameters are the same as those of Figure 3. Data plotted by dotted dark blue lines in the top and the middle graphs correspond to the condition, $V_{ex}<0$, i.e., a linear mode.

**Figure 5:**
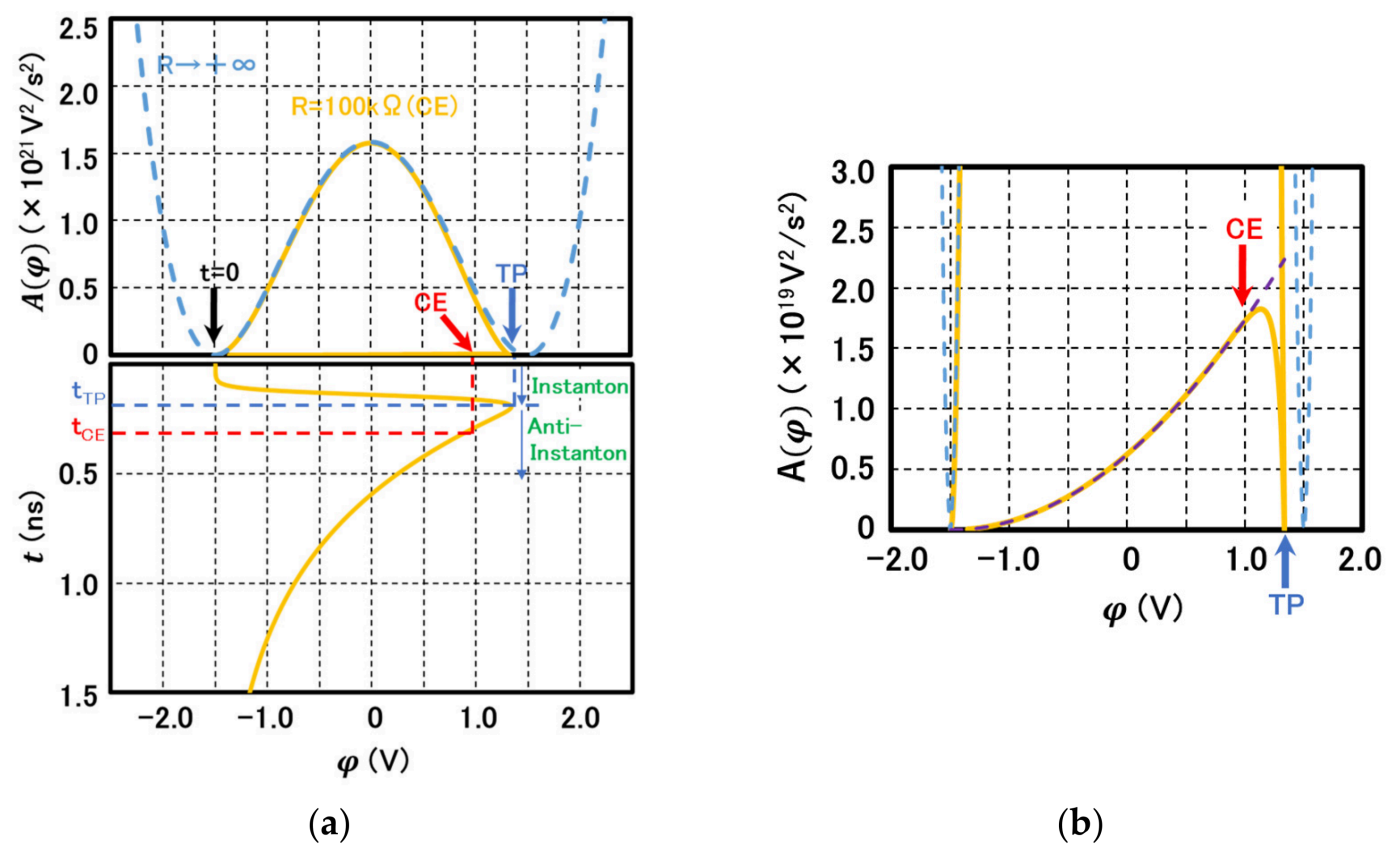
Calculated AEPs ($A\left(\varphi \right)$) for resistive quenching conditions. (**a**) (Top) Calculated $A\left(\varphi \right)$ plots of a resistive quenching with CE condition (solid yellowish line) and that of a capacitive quenching condition (dotted blue-line); (Bottom) φ vs. time for the same resistive quenching condition as the one on the top. The physical parameters are the same as listed in Table 1. (**b**) The same plot as (**a**) with the vertical scale magnified by about 100 times. The dotted purple line is a theoretical line calculated from (38). The red arrow pointing at φ≈1 V designates the position after which CE is in effect. The blue arrow points at around ~1.4 V designate the TP at which the sign of $\dot{\varphi }$ changes from positive to negative as indicated in Figure 3b.

**Figure 6:**
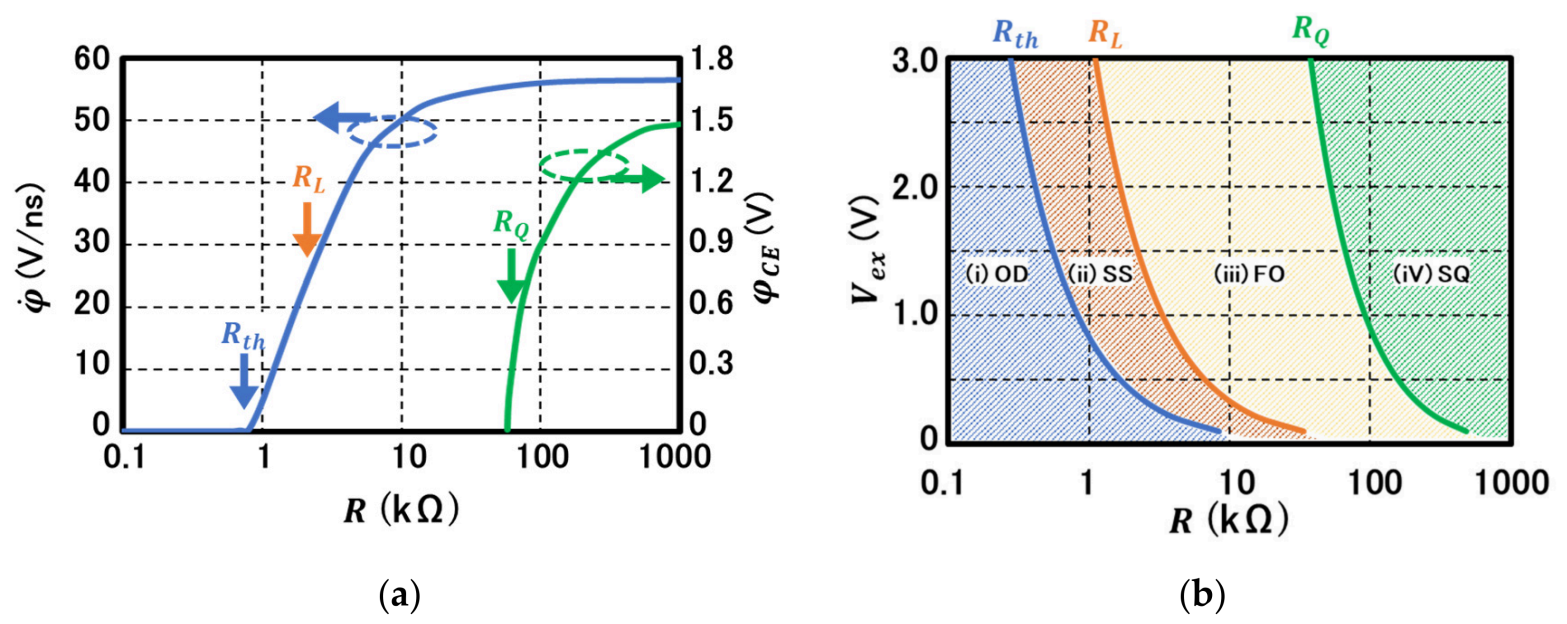
(**a**) Blue-colored plot (referenced to the left vertical axis): Calculated velocity term ($\dot{\varphi }$) at φ=0 as a function of QR value exhibiting a phase transition-like behavior at $R_{th}=$ 550 Ω indicated by a blue arrow. The other two transition points of quenching conditions, $R_{L}(=2.2\;k\Omega )$ (orange arrow) and $R_{Q}(\approx 70\;k\Omega )$ (green arrow), are also indicated; green-colored plot: carrier extinction voltage ($\varphi_{CE}$) relative to the BV as a function of the QR value referenced to the right vertical axis. $\varphi_{CE}$ emerges only for $R>R_{Q}$ showing also a phase transition-like behavior. With increase in the QR, $\varphi_{CE}$ also increases in accordance with the higher probability of successful quenching. The parameters are the same as those in Table 1. (**b**) $R-V_{ex}$ plane map of avalanche diode’s operation phases. The three boundary lines are calculated from (26) of [23] ($R_{th})$ (blue line); from (26) ($R_{L})$ (orange line) and from ($R_{Q}$) (9) (orange line). Each hatched area corresponds to each region classified in Section 6.1.

**Figure 7:**
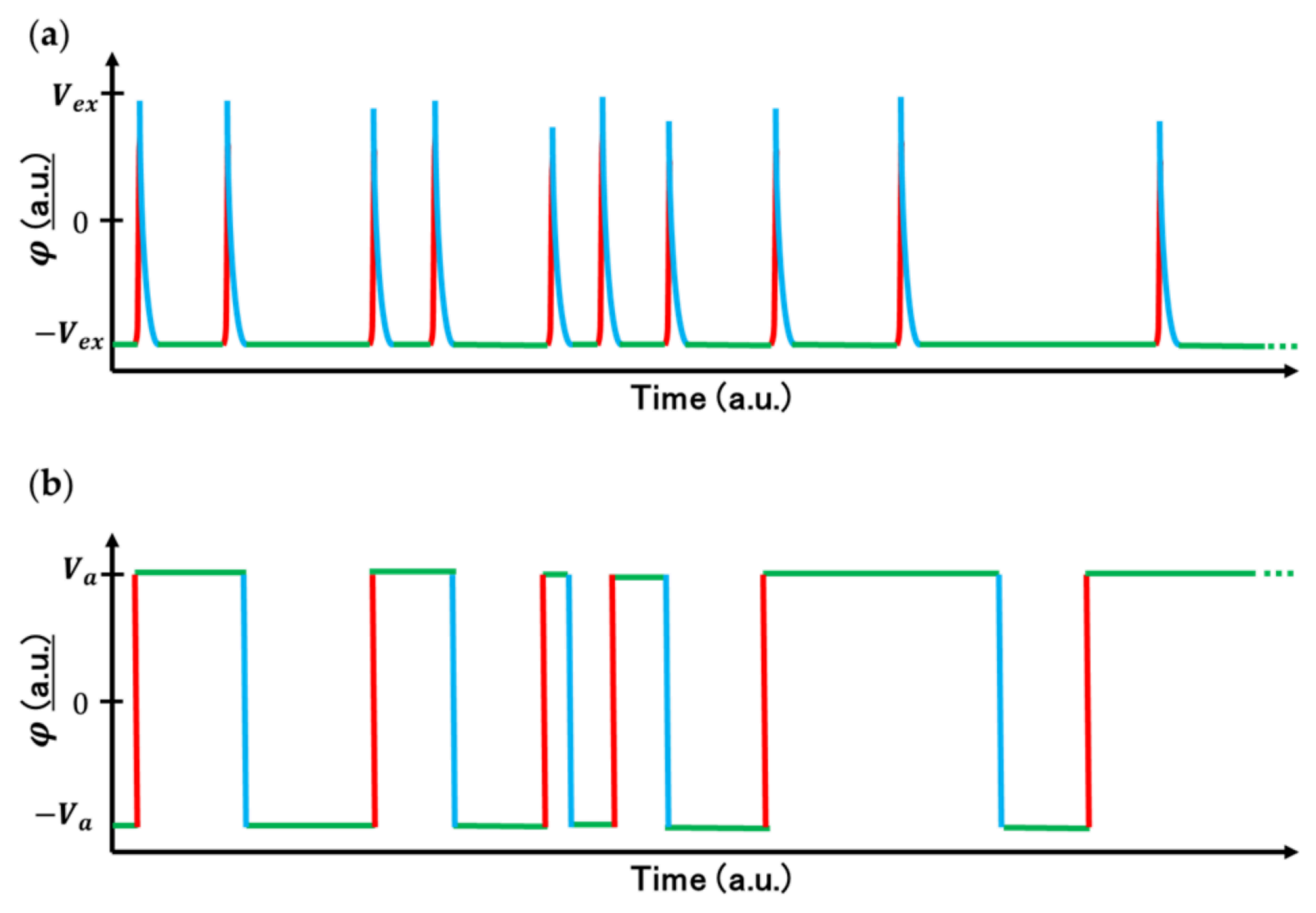
(**a**) An illustration of a sequence of successfully quenched GM-pulses predominantly located at the one of the double well bottom φ $=-V_{ex}$ (green lines) due to the paring of instanton (red lines)-anti-instanton (blue lines). (**b**) An illustration of random-telegraph noise due to random and alternate generation of instantons (red) and anti-instantons (blue) between hypothetical local minima (φ $=\pm V_{a}$) (green lines) showing the average value located at nearly origin.

**Table 1:** Definitions of symbols [23].

Symbol	Definition	Value
W	Width of depletion region	0.80 μm
C	Capacitance of the output node	6.0 fF
$v_{e}$	Electron saturation velocity	$1.02\times 10^{7}$ cm/s
$v_{h}$	Hole saturation velocity	$8.31\times 10^{6}$ cm/s
$\alpha_{0}$	Coefficients of impact ionization ratio	$3.80\times 10^{6}$ cm^−1^
$\beta_{0}$		$2.25\times 10^{7}$ cm^−1^
a		$1.75\times 10^{6}$ V/cm
b		$3.26\times 10^{6}$ V/cm
q	Elementary charge	$1.60\times 10^{-19}$ C

**Table 2:** Characteristics table for phases of excess-biased avalanche diode’s carrier dynamics.

Characteristics	(i) OD	(ii) SS	(iii) FO	(iv) SQ
Value of QR	$R<R_{th}$	$R_{th}<R<R_{L}$	$R_{L}<R<R_{Q}$	$R_{Q}<R$
Device Type	IMPATT Diode	-	-	SPAD
Stability of V_BD_	Stable	Initial: Unstable Final: Stable	Unstable	Unstable
Quenching	No.	No.	No.	Yes.
AEP Symmetry	Initial: Broken Final: Symmetric	Initial: Broken Final: Symmetric	Broken	Broken

**Table 3:** Analogy between superconductivity and APD operation modes.

Item	Superconductivity	APDs Operational Modes
Order Parameter	$\psi \left(r\right)$ *^1^	φ(t)
Evaluated Energy	$F_{n}+\alpha {\left|\psi \right|}^{2}+\frac{\beta }{2}{\left|\psi \right|}^{4}$	$A\left(\varphi \right)$
Symmetric Phase	Normal conductor	DC-biased regime
	$T>T_{C}$ *^2^	$R>R_{C}$
	$\psi_{min}=0$	$V_{ex}=0$
Symmetry-Broken Phase	Superconductive	SPAD quenching regime
	$T<T_{C}$	$R\geq R_{Q}$
	$\left|\psi_{min}\right|>0$	$V_{ex}>0$, $V_{TP}>0$
Wave Function	$\prod_{k}\left(u_{k}+v_{k}\cdot {c_{k↑}}^{†}\cdot {c_{-k↓}}^{†}\right)\left|\left.\Psi_{0}\right\rangle \right.$ *^3^	$A{\left\{{\left(1-P_{PDE}\right)}^{\frac{1}{2}}+K\cdot d^{†}\cdot b^{†}\right\}}^{n}\left|-\left.V_{ex},\;0\right\rangle \right.$ *^4^
Quasi-particle	Cooper Pair	Instanton–Anti-instanton pair
Paring Force	Electron–phonon interaction	Carrier Extinction (CE)

*^1^: pseudo-wave function describing spatial (coordinate r) density of electrons.; *^2^: Critical temperature of a superconductor; *^3^: BCS ground state wave function where $u_{k}$ and $v_{k}$ are complex numbers and ${u_{k}}^{2}+{v_{k}}^{2}=1.$ ([26], pp. 110–147); *^4^: Equation (60) where $K=\sqrt{\eta }\cdot \sqrt{\kappa }\cdot \exp\left(i\theta \right).$

## Data Availability

The original contributions presented in this study are included in the article. Further inquiries can be directed to the corresponding author.

## Abbreviations

The following abbreviations are used in this manuscript:

AEP	available energy profile
ATP	avalanche triggering probability
BV	breakdown voltage
CE	carrier extinction
EV	excess voltage
FO	Fluctuation/Oscillation
FP	final point
GM	Geiger mode
IMPATT	Impact Ionization Avalanche Transit-Time (diode)
IP	initial point
LE	Lienard equation
MR	multiplication region
OD	overdamped
QR	quenching resistance
SPAD	single-photon avalanche diode
SQ	successful quenching
SS	spiral sink
TP	turning point

## Appendix A. Quantization of the Lienard Equation

In this section, quantization conditions of the Lienard equation are derived. Let ψ be a function of time satisfying the classical Lienard equation (10);

(A1) $$\ddot{\psi }+f\left(\psi \right)\cdot \dot{\psi }+g\left(\psi \right)\cdot \psi =0.$$

According to (14) and (15), the Hamiltonian of (A1) is obtained as

(A2) $$H\left(\psi ,\;\dot{\psi }\right)=\frac{1}{2}{\;\dot{\psi }}^{2}+\int_{\psi_{0}}^{\psi }f\left(y\right)\cdot \dot{y}\cdot dy+\int_{\psi_{0}}^{\psi }g\left(y\right)\cdot y\cdot dy,$$

which leads to the Hamilton Equation (20)

(A3) $$\frac{\partial H}{\partial \psi }=-\ddot{\psi },\;\;\;\frac{\partial H}{\partial \dot{\psi }}=\dot{\psi },$$

indicating that ψ and $\dot{\psi }$ are conjugate variables. To promote these variables as quantized fields, firstly, we treat them as field operators and impose the following usual commutator relations.

(A4) $$\left[\psi ,\dot{\psi }\right]=-i\hbar ,$$

(A5) $$\left[\psi ,\psi \right]=0,\;\;\left[\dot{\psi },\dot{\psi }\right]=0.$$

If ψ and $\dot{\psi }$ satisfy the quantum mechanical Heisenberg equation of motion, they are shown to be quantized. As for ψ, it is shown as

(A6) $$\left[\psi ,H\right]=\frac{1}{2}\left(\psi {\dot{\psi }}^{2}-{\dot{\psi }}^{2}\psi \right)=i\hbar \dot{\psi }.$$

The first equality is due to the fact that the second and the third terms of the right-hand-side of (A2) are functions of ψ and due to the first equation of (A5). The second equality follows from (A4). As for $\dot{\psi }$, we first define the second and third terms of (A2) as

(A7) $$F\left(\psi \right)\equiv \int_{\psi_{0}}^{\psi }f\left(y\right)\cdot \dot{y}\cdot dy,\;\;G\left(\psi \right)\equiv \int_{\psi_{0}}^{\psi }g\left(y\right)\cdot y\cdot dy.$$

We also note that, by successive use of (A4) and by induction, the following identity holds

(A8) $$\dot{\psi }\psi^{n}-\psi^{n}\dot{\psi }=-ni\hbar \psi^{n-1}.$$

Now, the Heisenberg equation for $\dot{\psi }$ is as follows:

(A9) $$\left[\dot{\psi },H\right]=\dot{\psi }F\left(\psi \right)-F\left(\psi \right)\dot{\psi }+\dot{\psi }G\left(\psi \right)-G\left(\psi \right)\dot{\psi }.$$

If $F\left(\psi \right)$ and $G\left(\psi \right)$ are polynomial functions of ψ or analytic in ψ, then (A9) leads to

(A10) $$\left[\dot{\psi },H\right]=-i\hbar \left(F^{\prime }\left(\psi \right)+G^{\prime }\left(\psi \right)\right)=-i\hbar \left(f\left(\psi \right)\cdot \dot{\psi }+g\left(\psi \right)\cdot \psi \right)=i\hbar \ddot{\psi },$$

satisfying the Heisenberg equation. The last equality of (A10) recovers the Lienard equation. Thus, the Lienard equation plays the role of the wave equation in the quantum mechanical system.

To summarize, the classical Lienard equation is quantized by imposing the commutator relations (A4), (A5) and under the condition that $F\left(\psi \right)$ and $G\left(\psi \right)$ are polynomial functions of ψ or analytic in ψ with ψ and $\dot{\psi }$ being canonical conjugate variables. By following the above derivation in the inverse direction, it is seen that commutator relations (A4) and (A5) hold. Thus, (A4), (A5) and the conditions of $F\left(\psi \right)$ and $G\left(\psi \right)$ being analytic or polynomials are necessary and sufficient conditions for the “present” Lienard equation to be quantized. In general, quantization of dissipative systems requires the construction of a specific Hamiltonian [44]. However, the present case of the Lienard equation is a special case that such restrictions are released. The following factors are considered be major reasons: (i) All the relevant energy terms are contained in the Hamiltonian and the total energy is shown to be conserved as (19). (ii) Rewriting the Lienard equation as $\ddot{\psi }=-\partial V\left(\psi \right)/\partial \psi$ indicates that the field is described as a conservative force field. It is noted that other type of the Lienard equation, the quantum mechanical Van der Pol oscillator, has recently been reported under intensive studies [44,45]. (10) is a new type of Lienard equation also leading to the quantization conditions above. The fact that such physics is found in the commercially or industrially important device, SPAD, is emphasized.

## Appendix B. Outline of Derivation of the Feynman-Kernel Solution

In this section, the eigenvalue problem of the capacitive quenching process described by the Feynman kernel is shown to be equivalent to that of the instanton solved by the Euclidean kernel as in [34,35]. The general form of the eigenvalue equation is

(A11) $$\left\{-\frac{d^{2}}{dt^{2}}-V^{\prime \prime }\left(\varphi_{C}\right)\right\}\cdot u_{a}\left(t\right)=\lambda_{a}\cdot u_{a}\left(t\right),$$

where $V^{\prime \prime }\left(\varphi_{C}\right)$ is a second derivative of a potential function evaluated at $\varphi =\varphi_{C}$, and $u_{a}\left(t\right)$ is an eigenfunction with an eigenvalue $\lambda_{a}$ indexed by a suffix a. Substituting (45) into $V\left(\varphi \right)$ and differentiating it twice with respect to φ, and then substituting (36) into φ as the classical solution $\varphi_{C}$, we obtain

(A12) $$\left[-\frac{d^{2}}{dt^{2}}+\frac{1}{2{\tau_{Q}}^{2}}\left\{3\tanh^{2}\left(t/(2\tau_{Q})\right)-1\right\}\right]\cdot u_{a}\left(t\right)=\lambda_{a}\cdot u_{a}\left(t\right),$$

which can be shown to be identical in form to (23) of [34] or (9.241) of ([32], pp. 403–416). Therefore, as far as the eigenvalues and therefore determinant values are concerned, the capacitive quenching process described by the Feynman kernel is equivalent to the instanton problem solved by the Euclidean kernel. Apart from normalization constants, complete solutions of (A12) are known as follows ([32], pp. 403–416).

(i) Zero mode denoted by a=0 with $\lambda_{0}=0$ and $u_{0}\left(t\right)=1/\cosh^{2}\left(t/(2\tau_{Q})\right)$.
(ii) Discrete mode denoted by a=1 with $\lambda_{1}=3/\left(2{\tau_{Q}}^{2}\right)$ and $u_{1}\left(t\right)=\tanh\left(t/(2\tau_{Q})\right)\cdot \mathrm{sech}\left(t/(2\tau_{Q})\right)$.
(iii) Continuous mode denoted by a continuous variable a=p with $\lambda_{p}=p^{2}+2/{\tau_{Q}}^{2}$ and $u_{p}\left(t\right)=\exp\left(i\cdot pt/\sqrt{2}\right)\cdot \left[3\tanh^{2}\left(t/(2\tau_{Q})\right)-1-2p^{2}{\tau_{Q}}^{2}-i\cdot 3\sqrt{2}p\tau_{Q}\cdot \tanh\left(t/(2\tau_{Q})\right)\right]$.

As for the determinant, referencing a determinant of the harmonic oscillator, (42) can be written as

(A13) $$G\left(V_{f},\;T,V_{i},\;0\right)=\frac{{\left[det^{\prime }\left\{i\cdot \left(-\frac{d^{2}}{dt^{2}}-V^{\prime \prime }\left(\varphi_{C}\right)\right)\right\}\right]}^{-1/2}}{{\left[det\left\{i\cdot \left(-\frac{d^{2}}{dt^{2}}+\omega^{2}\right)\right\}\right]}^{-1/2}}\cdot {\left[det\left\{i\cdot \left(-\frac{d^{2}}{dt^{2}}+\omega^{2}\right)\right\}\right]}^{-\frac{1}{2}}\cdot e^{\frac{iS_{C}}{\hbar }}\cdot N.$$

For the discrete and continuous modes, the factors due to the imaginary unit are canceled in the first term due to the equal contributions from the denominator and from the numerator. The contribution of the imaginary unit in the second term is also only to the phase factor. Since the eigenvalue and the eigenfunction of the zero mode are identical to those obtained in [34], the boundary condition $u_{0}\left(t\to \pm \infty \right)$ = 0. The zero-mode results can also be preserved. Thus, the amplitude of the pre-factor (coefficient of $e^{\frac{iS_{C}}{\hbar }}$) is identical to that described in [34].

## Appendix C. Derivation of the Avalanche Triggering Probability Term in the Green’s Function

In Section 5.1, a general form of the Green’s function for a quantum state is given as (42). In general, calculation is quite long. Fortunately, in the limiting cases of the capacitive quenching regime and the IMPATT operation limit, analytical solutions to the identical problem are available in the literature [34,35], except that these solutions are calculated with the Euclidian kernel. Here, we wish to obtain the form with the Feynman kernel. We pointed out that the difference between the two schemes is only a phase factor of a free particle term. In the following, we will confirm this statement by explicitly calculating the initial condition term of the instanton representing the avalanche triggering probability distribution.

We restate the initial condition of the GM-pulse generation as follows; the system initially (at t=0) located at $\varphi =-V_{ex}$ evolves to the same state, $\varphi =-V_{ex}$, at the end of time (t=T). Noting that $-V^{\prime \prime }\left(\varphi_{C}\right)=-V^{\prime \prime }\left(-V_{ex}\right)=1/{\tau_{Q}}^{2}$, for both limiting cases of the capacitance quenching regime and the IMPATT regime, the Green’s function is then described as

(A14) $$G\left(-V_{ex},\;T,-V_{ex},\;0\right)=e^{\frac{iS_{C}}{\hbar }}\cdot N\cdot {\left[det^{\prime }\left\{i\cdot \left(-\frac{d^{2}}{dt^{2}}+\frac{1}{{\tau_{Q}}^{2}}\right)\right\}\right]}^{-1/2},$$

where a classical solution is set at a constant as $\varphi_{C}=-V_{ex}$. (A14) simply describes a probability amplitude that the system initially located at the top of the left hill will stay at the same place, i.e., $\varphi =-V_{ex}$, at the end as illustrated as a black line circle in the bottom of Figure 4. Once (A14) is given, setting $1/\tau_{Q}\to 0$ leads to a representation of a free particle without any bound term, i.e.,

(A15) $$G_{FP}\left(-V_{ex},\;T,-V_{ex},\;0\right)=e^{\frac{iS_{C}}{\hbar }}\cdot N\cdot {\left[det^{\prime }\left\{i\cdot \left(-\frac{d^{2}}{dt^{2}}\right)\right\}\right]}^{-1/2},$$

where FP indicates the expression represents a free particle. On the other hand, a standard expression for a (unit mass) free particle with the Feynman kernel is readily available as $\sqrt{1/\left(2\pi T\cdot i\cdot \hbar \right)}$, where i is an imaginary unit and ℏ is the Planck’s constant ([27], pp. 210–219). This term should be identical to (A15). Therefore, we obtain an expression for (A14) by taking a ratio of (A14) and (A15) and by multiplying the standard Feynman kernel expression as

(A16) $$G\left(-V_{ex},\;T,-V_{ex},\;0\right)=\frac{{\left[det^{\prime }\left(-\frac{d^{2}}{dt^{2}}+\frac{1}{{\tau_{Q}}^{2}}\right)\right]}^{-1/2}}{{\left[det^{\prime }\left(-\frac{d^{2}}{dt^{2}}\right)\right]}^{-1/2}}\cdot \sqrt{\frac{1}{2\pi T\cdot i\cdot \hbar }}.$$

Now, we note that the first term of the left-hand side is identical to the factor calculated with the Euclidean kernel in [34,35] whereas the second term is the standard free particle term of the Feynman kernel with the only difference to that of the Euclidian kernel being the presence of the imaginary unit contributing only to a phase factor. Thus, our previous statement is confirmed. By noting that the *n*-th eigenvalue of the operator $\left(-d^{2}/{dt}^{2}+1/{\tau_{Q}}^{2}\right)$ is $\left({\left(n\pi /T\right)}^{2}+1/{\tau_{Q}}^{2}\right)$ with the eigenfunction $c_{n}\cdot \sin\left(n\pi \cdot t/T\right)$ (where $c_{n}$ is a constant), and taking the limit, $1/\tau_{Q}\to 0$, in the denominator, the final expression is obtained as [34,35]

(A17) $$G\left(-V_{ex},\;T,-V_{ex},\;0\right)=\frac{{\left[\prod_{n=1}^{\infty }\left({\left(n\pi /T\right)}^{2}+1/{\tau_{Q}}^{2}\right)\right]}^{-1/2}}{{\left[\prod_{n=1}^{\infty }\left({\left(n\pi /T\right)}^{2}\right)\right]}^{-1/2}}\cdot \sqrt{\frac{1}{2\pi T\cdot i\cdot \hbar }}={\left(\frac{1/\tau_{Q}}{\sinh\left(T/\tau_{Q}\right)}\right)}^{\frac{1}{2}}\cdot \sqrt{\frac{1}{2\pi \cdot i\cdot \hbar }}\;,$$

where an identity, $\prod_{n=1}^{\infty }\left(1+{\left(y/n\right)}^{2}\right)=\{\sinh\left(\pi y\right)\}/(\pi y)$, is utilized in the second equality [46,47]. We reemphasize that the expression is the same for both the capacitive quenching regime and the IMPATT regime.
